# Targeted Isolation of Antibiotic Brominated Alkaloids from the Marine Sponge *Pseudoceratina durissima* Using Virtual Screening and Molecular Networking

**DOI:** 10.3390/md20090554

**Published:** 2022-08-29

**Authors:** James Lever, Florian Kreuder, Jason Henry, Andrew Hung, Pierre-Marie Allard, Robert Brkljača, Colin Rix, Aya C. Taki, Robin B. Gasser, Jan Kaslin, Donald Wlodkowic, Jean-Luc Wolfender, Sylvia Urban

**Affiliations:** 1 School of Science (Applied Chemistry and Environmental Sciences), RMIT University, GPO Box 2476 Melbourne, VIC 3001, Australia; 2 Australian Regenerative Medicine Institute, Monash University, Clayton, VIC 3800, Australia; 3 Neurotoxicology Lab., School of Science (Biosciences), RMIT University, Bundoora, VIC 3083, Australia; 4 Department of Biology, University of Fribourg, 1700 Fribourg, Switzerland; 5 Monash Biomedical Imaging, Monash University, Clayton, VIC 3168, Australia; 6 Department of Veterinary Biosciences, Melbourne Veterinary School, Faculty of Veterinary and Agriculture Sciences, The University of Melbourne, Parkville, VIC 3010, Australia; 7 School of Pharmaceutical Sciences of Western Switzerland, University of Geneva, CMU, Rue Michel-Servet 1, 1211 Geneva, Switzerland; 8 Institute of Pharmaceutical Sciences of Western Switzerland, University of Geneva, CMU, 1211 Geneva, Switzerland

**Keywords:** MRSA pathogen, virtual screening, in silico molecular docking, targeted anti-biotic isolation, zebrafish, cheminformatic analysis, sponge metabolites, chemical space topology, molecular networking

## Abstract

Many targeted natural product isolation approaches rely on the use of pre-existing bioactivity information to inform the strategy used for the isolation of new bioactive compounds. Bioactivity information can be available either in the form of prior assay data or via Structure Activity Relationship (SAR) information which can indicate a potential chemotype that exhibits a desired bioactivity. The work described herein utilizes a unique method of targeted isolation using structure-based virtual screening to identify potential antibacterial compounds active against MRSA within the marine sponge order Verongiida. This is coupled with molecular networking-guided, targeted isolation to provide a novel drug discovery procedure. A total of 12 previously reported bromotyrosine-derived alkaloids were isolated from the marine sponge species *Pseudoceratina durissima,* and the compound, (+)-aeroplysinin-1 (**1**) displayed activity against the MRSA pathogen (MIC: <32 µg/mL). The compounds (**1**–**3**, **6** and **9**) were assessed for their central nervous system (CNS) interaction and behavioral toxicity to zebrafish (*Danio rerio*) larvae, whereby several of the compounds were shown to induce significant hyperactivity. Anthelmintic activity against the parasitic nematode *Haemonchus contorutus* was also evaluated (**2**–**4**, **6**–**8**).

## 1. Introduction

*Staphylococci* infections are a major concern for hospitals world-wide with infections occurring primarily in people who are immunocompromised, generally because of recent surgery, especially in patients who experience amputations, open wounds, or ongoing chemotherapy treatments. This problem has been exacerbated by the persistent threat of antibiotic resistance displayed by this bacterium. The World Health Organization (WHO) has reported the mortality rate for people who display infection from the methicillin-resistant *Staphylococcus aureus* (MRSA) is 64% higher than those infected with the non-resistant strains of *S. aureus* [1]. Methicillin resistance was observed in these organisms in 1961, only two years after the medical community switched to the use of methicillin upon observing the bacterium’s resistance to penicillin [2]. Methicillin resistance manifests itself in MRSA strains via the production of an additional Penicillin Binding Protein (PBP) within the external membrane of the organism. Conventional treatment with the *β*-lactam antibiotics (penicillin, methicillin, oxacillin, etc.) would ordinarily see binding of these antibiotics with the PBPs, and result in the inhibition of the production of cross-linked peptidoglycans which thus reduces the structural integrity of the bacterial cell wall causing a critical failure of the cell to contain the cytoplasmic contents. In the case of MRSA an additional PBP exists, namely PBP2a, which shares many features with the other PBPs but exhibits very low affinity towards the *β*-lactam antibiotics, resulting in reduced efficacy of these drugs against MRSA strains [3]. The progressive development of bacterial resistance towards the standard plethora of antibiotics is cause for concern and has created significant motivation for the investigation of new structurally diverse antibiotics as well as in the search for novel mechanisms of action.

The marine environment has for decades provided a source of novel bioactive and structurally diverse natural products (NPs), many of which are currently being pursued within the pre-clinical pharmaceutical pipeline [4,5,6,7]. As well as possessing highly diverse metabolomes, marine invertebrates produce compounds with inherent bioavailability, some of which can be considered to have the property of drug-likeness, making them ideal candidates for the discovery of lead compounds against organisms such as drug resistant *S. aureus* [8].

Marine sponges of the order Verongiida produce a diverse array of antimicrobial brominated alkaloids that exhibit bioactive properties [4,6,9,10,11]. These brominated alkaloids have been proposed to be produced as a chemical defense against predation [12,13] as well as for their antifouling properties [14,15]. This was supported when Verongiida sponges were reported to actively secrete these compounds as a wound-induced antimicrobial protection after predation events [16,17]. While the antibiotic potential of these compounds has been reported, thus far, this line of investigation has been overshadowed by the significant promise of these compounds as anticancer agents [18,19,20,21]. The most widely studied genus of this order is *Pseudoceratina* Carter, 1885, which has, to date, yielded approximately 230 different brominated alkaloids from species within this genus [9,22]. Although many of these isolated compounds have shown promise in anticancer assessments, the majority have yet to be assayed against drug-resistant strains of bacteria, prompting a need for a further targeted study.

Targeted isolation of NPs is generally guided by lead compound characteristics derived from Structure Activity Relationship (SAR) studies. In many cases, however, documented SARs and even biological assay data either do not exist or are insufficient to guide the isolation of NPs. This poses a problem when prioritizing the isolation of biologically active NPs, as it is simply not feasible to isolate and assay all available NPs in an organism. As a result, in silico structure-based and ligand-based virtual screening, together with cheminformatics methodologies, have been adopted as alternative strategies to predict the biological activity of potential target compounds [23].

Virtual screening methods combined with the use of dereplication methodologies such as Molecular Networking (MN) [24,25,26] for targeted isolation of NPs appears to be an adapted combination for identifying the chemical composition of organisms, as well as for targeting bioactive constituents of these organisms for isolation and biological assessment. Computational virtual screening methodologies, combined with dereplication methodologies, could potentially provide a rapid, low-cost solution to conventional high-throughput targeted isolations. This type of analysis, utilizing predictive modelling and molecular docking affinity, can be geared towards discovering suitable ligands against a variety of targets with varying mechanisms of action.

This study presents a method for the targeted isolation of compounds based on chemical space topology, in a situation where literature bioactivity data is insufficient and too variable to produce a highly predictive SAR model. Chemical space topology combined with in silico molecular modelling provides an appropriate methodology to identify a short list of desirable compounds with high chemical similarity to known active compounds, as well as high binding affinity to known target proteins for MRSA. This virtual screening method resulted in the identification of several previously reported brominated alkaloids with potential bioactivity being included among the following: aeroplysinin-1 (**1**) [27], aerothionin (**2**) [27], homoaerothionin (**3**) [27], 17-deoxyfistularin-3 (**4**) [28], 11,17-deoxyfistularin-3 (**5**) [28], 2-(3,5-dibromo-1-hydroxy-4,4-dimethoxycyclohexa-2,5-dien-1-yl)acetamide (**6**) [29], 2-(3,5-dibromo-1-hydroxy-4,4-dimethoxycyclohexa-2,5-dien-1-yl)acetonitrile (**7**) [30], 2-(3,5-dibromo-2-hydroxy-4-methoxyphenyl)acetonitrile (**8**) [31], subereaphenol A (**9**) [32], subereaphenol B (**10**) [27], araplysillin I (**11**) [33] and subereamolline C (**12**) [34].

## 2. Results and Discussion

### 2.1. Data Set Curation and Treatment

**Figure 1:**
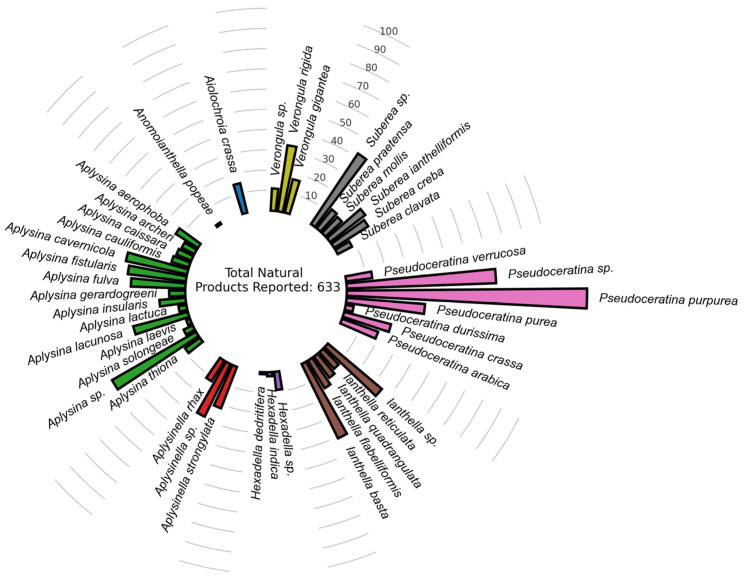
Primary dataset distribution of NPs previously reported for Verongiida sponges (Supplementary Materials).

In a previous publication by our research group which attempted to systematize the diversity and pharmaceutical potential of Verongiida sponges, we created a dataset of 633 NPs that had been previously reported from species within the Verongiida order of marine sponges [9], Figure 1.

This database was assessed using bipartite, scaffold and chemical similarity networks to explore both the chemical diversity across the taxonomy of this order, and to provide insight into the most ideal chemotypes for isolation, as measured by common pharmacokinetic properties such as drug-likeness, cLogP, LogS, MW, nHBAcc, nHBDon, nRotB, TPSA as well as predicted toxicity risks using the OSIRIS property explorer [35]. This database was used as the starting point for the current study reported herein.

Sponges of the order Verongiida appear to have many species that share brominated alkaloid NPs with their inter-genus counterparts. In particular, the genus *Pseudoceratina* exhibits an interesting trait regarding its metabolite distribution. The NPs produced by *Pseudoceratina* display a diverse set of scaffolds indicating a complex biosynthetic chemistry. Further, of all the genera within the order Verongiida, *Pseudoceratina*, has the largest number of scaffolds derived from their reported NPs [9]. The high prevalence of different scaffolds produced by marine sponges from the genus *Pseudoceratina* makes this an ideal candidate for targeted isolation using virtual screening.

The Verongiida NPs dataset was cross-checked with the ChEMBL database for reports of previous activity against both *S. aureus* as well as MRSA strains yielding various types of reported activity (Inhibition zone, MIC, IC_50_ and % inhibition) for a subset of 96 of the original 633 compounds, however, the remaining 537 NPs showed no recorded activity against either variety of bacteria. This subset of 96 was attributed as the ChEMBL actives subset.

### 2.2. Docking-Based Virtual Screening

All 633 compounds from the Verongiida dataset were screened using in silico molecular docking against 19 selected MRSA targets. Targets were selected that encompassed a variety of inhibitory modalities including cell division, DNA replication, glycolysis, protein modification, protein secretion, peptidoglycan synthesis, cellular regulation, fatty acid synthesis and stress response. Targets were selected from two sources (i) previous virtual screening studies searching for common natural products that may have strong interaction with *Staphylococci* targets and (ii) review articles focusing on novel targets for antibiotics treatment of *Staphylococci* infections [36,37,38,39,40]. Amongst the targets used some have been used as MOAs for clinically approved drugs treating MRSA including mupirocin (Isoleucyl-tRNA synthetase) [41] and ozenoxacin (DNA Gyrase subunit A) [42] whilst many others have been used in the clinical trial phase [39]. Docking affinity data was collected by docking all 633 reported NPs against the 19 selected targets to assess which compounds showed high potential binding affinity with these targets (Table 1).

**Figure 2:**
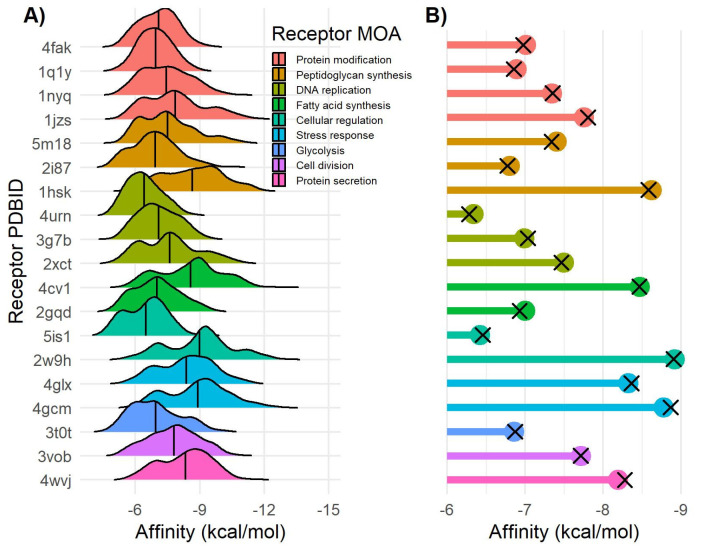
Docking affinity of Verongiida NPs data set. (**A**) Density distribution of affinity values for each MRSA target. (**B**) Lollipop plot representing mean value of *Pseudoceratina* NPs subset (colored dots) and Verongiida subset displaying activity against *Staphylococcus* pathogens in ChEMBL (black cross).

Affinity values for the Verongiida data set displayed the highest mean affinities against Mur B (1hsk) −8.65 kcal/mol, FabI (4cv1) −8.57 kcal/mol, DHFR (2w9h) −8.98 kcal/mol, LigA (4glx) −8.38 kcal/mol, TrxB (4gcm) −8.90 kcal/mol and SpsB (4wvj) −8.33 kcal/mol (Figure 2A). In many of these cases skewing was observed, indicating that a large proportion of the compounds within the dataset displayed significantly higher affinity than the overall mean. Interestingly, much lower mean affinity data was observed against targets that acted via protein modification, glycolysis or DNA replication with MOAs, often skewing toward lower affinity values, suggesting these to potentially be less desirable targets for this dataset. A subset of the data was made that included only compounds previously isolated from the genus *Pseudoceratina* which was compared against the ChEMBL actives subset (Figure 2B). 

Notably, there was very little difference between the mean affinity values of these datasets, and indeed between the mean affinity of the Verongiida data set, indicating a consistency in docking variability across all three datasets. Docking affinity can show wide variability between ligands, with even the smallest change in chemical motifs producing different affinity scores, due to the increased likelihood of ligands interacting with different residues within the binding pocket of the target enzyme. In order to achieve a relatively even comparison of affinity scores, and to allow diversity to persist throughout the screening process, the compounds were grouped into structural motif classes. This was achieved using chemical space similarity networks. Chemical space networking is usually used to observe clustering of highly similar compounds within common chemotype clusters.

All compounds were represented as nodes within these networks with edges linking the compounds together if two conditions were met: i) the compounds displayed the same Murcko scaffold [60] structure; indicating them to be of a similar general chemotype, and ii) the compounds achieved at least a 0.5 Tanimoto correlation coefficient when comparing their Morgan fingerprints; indicating the variation within the general chemotype to be sufficiently small for comparison of binding affinity values. Compounds that did not achieve any edges under these conditions (singletons) were removed from the network as they would not provide any useable clustering information. In total 504 compounds of the original 633 compounds were included in the final structural similarity networks.

**Figure 3:**
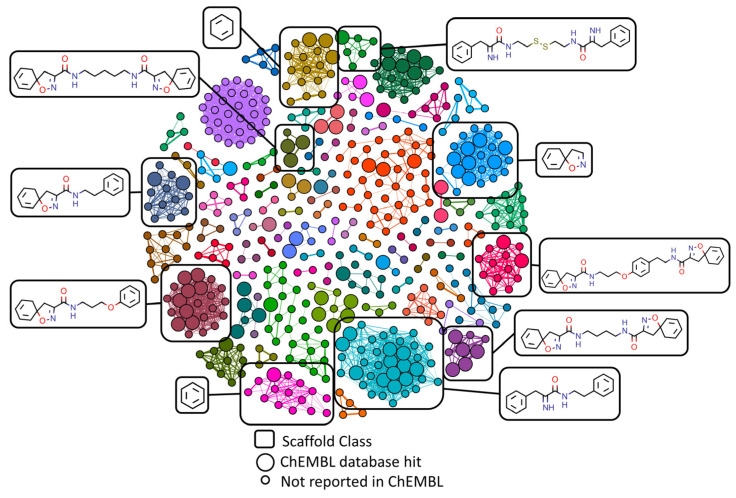
Similarity networking of Verongiida dataset against actives from ChEMBL database. Clustering applied to network illustrating the scaffolds representing the major clusters. Enlarged nodes representing the compounds that were reported as active against either *Staphylococcus aureus* or MRSA within ChEMBL database.

Separation of the main type of chemical compound classes within the Verongiida data set was achieved with Louvain clustering [61], however, due to the nature of network construction, there were no inter-cluster links. Networks constructed in this manner will allow for two separate clusters to have the same scaffold by design. This is due to compounds being structurally the same when comparing their central scaffolds but having a large amount of variance when considering their aliphatic portions as well as their different functional groups. Clustering was achieved such that all varieties of brominated alkaloids were separated into clusters representative of only one type of scaffold where all major compound classes were observed; mono- and bis- spiroisoxazolines, bastadins, dibromocyclohexadienes, verongiabenzenoids, bromotyramines both with oxime functionality and without, psammaplins, amongst others, Figure 3.

The ChEMBL actives data subset was used to identify which clusters contain compounds within them that have shown activity previously, making them the basis for determining which chemotypes are of interest. Compounds that exist within the same cluster as an ‘active’ but have not yet been assayed according to the ChEMBL database were of interest for virtual screening using docking affinity, Figure 3. Clustered communities that include at least one active totaled 27 (301 compounds including actives) of the original 84 communities (504 compounds).

**Figure 4:**
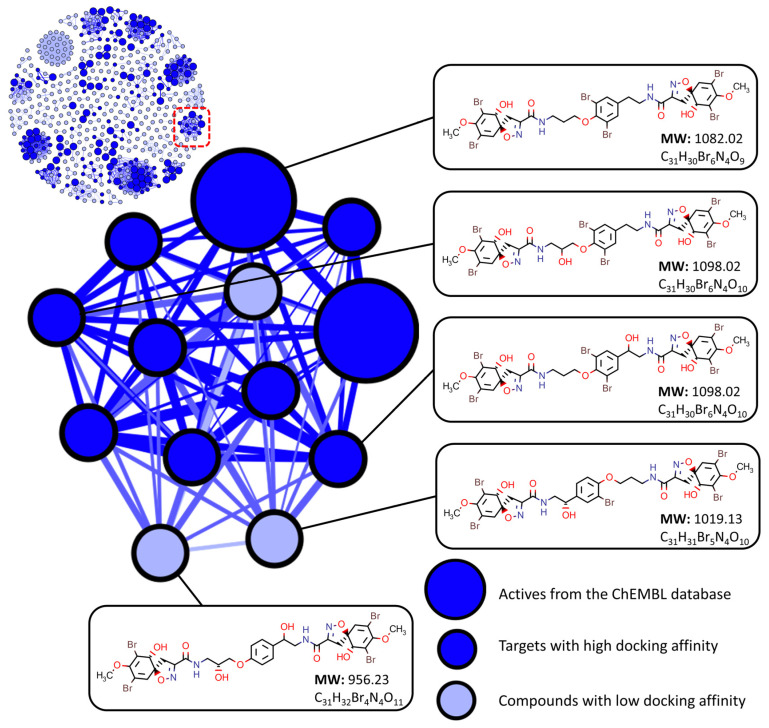
Target screening using similarity networking and molecular docking affinity scoring. Compounds are considered to have low docking affinity if they do not have an affinity score within the top 10 percent of values for at least one target receptor when compared to their cluster counterparts.

Docking affinities were then compared within each cluster containing an active to determine which compounds would be the best candidates for targeted isolation. For a compound to be selected as a suitable target, the following conditions needed to be met: (i) compounds must be within the same cluster as an active, and (ii) compounds must be within the top ten percent of affinity values for at least one target enzyme when compared to other compounds within their respective cluster, Figure 4. This method of screening left 192 (including actives) candidates for isolation and assay, removing 109 compounds due to poor affinity with all selected targets.

As compounds were only assessed on their affinity performance against their closely related structural derivatives this resulted in a list of candidates where structural diversity was preserved. Compounds from a variety of different structure classes and chemical scaffolds were selected in the final target list and examples from some of the larger clusters are shown in Table 2. 

### 2.3. Molecular Networking and Compound Isolation

Four marine sponge specimens were collected via SCUBA just offshore at Queenscliffe, Port Phillip Bay, Victoria, Australia at a depth of 1.5–3 m on 23 March 2016. Profiling of the four marine sponge specimens (2018_59, 2018_61, 2018_62 and 2018_63) was performed using data dependent UHPLC-MS experiments run in the positive ionization mode. These data were constructed into a Feature-Based Molecular Network (FBMN) using the Global Natural Products Social Molecular Networking (GNPS) platform [24,62] representing MS/MS metabolite profiles of all four marine sponges within the same network. Specimens of the sponges were initially extracted using a solvent system of 3:1 MeOH:DCM and then subsequently sequentially solvent partitioned (triturated) into a DCM soluble fraction and a MeOH soluble fraction for each specimen. All 8 extracts were analyzed using UHPLC-MS and data pre-treatment was performed using MZmine 2.53 [63]. The MS data was then uploaded to the GNPS platform creating a composite network using the FBMN workflow including data from all 8 extracts in the final network. The composite FBMN consisted of 4857 individual nodes representing features from the MS/MS data. Spectral comparisons of the GNPS libraries resulted in 134 annotations of which only 3 brominated alkaloids were annotated (Purealidin M, Purealidin N, Purealidin X). As a result of the low number of brominated alkaloid annotations, an in silico database (ISDB) annotation strategy was adopted [64]. This strategy utilized organism taxonomy as well as consensus chemical pathway information for compounds within the same cluster to orient the metabolite annotation process [65]. The result was a prioritization of compound annotations originating from organisms with low taxonomic distance to that of the *Pseudoceratina* specimens as well as prioritizing compound consensus within each cluster of the FBMN. Specific isotopic pattern observed in the MS1 spectra was also used to assist annotation as a large proportion of compounds produced by these sponges are highly brominated. This yielded putative annotations for 205 features (nodes) representing 156 unique compounds. Some nodes present within the FBMN represent adducts or even isotopes meaning that not every node present represents a single unique compound. Thus, in this case, some compounds can be represented by several nodes which is to be expected for these extracts given the high number of brominated compounds present. Upon inspection of the taxonomically prioritized annotations, only 36 compounds were brominated alkaloids, with 6 compounds being from the shortlist of target candidates created from chemical similarity networking and docking affinity calculations (Supplementary Materials Table S1 [10,17,66,67,68,69,70,71,72,73,74,75,76,77,78,79,80,81,82,83,84,85,86]).

**Figure 5:**
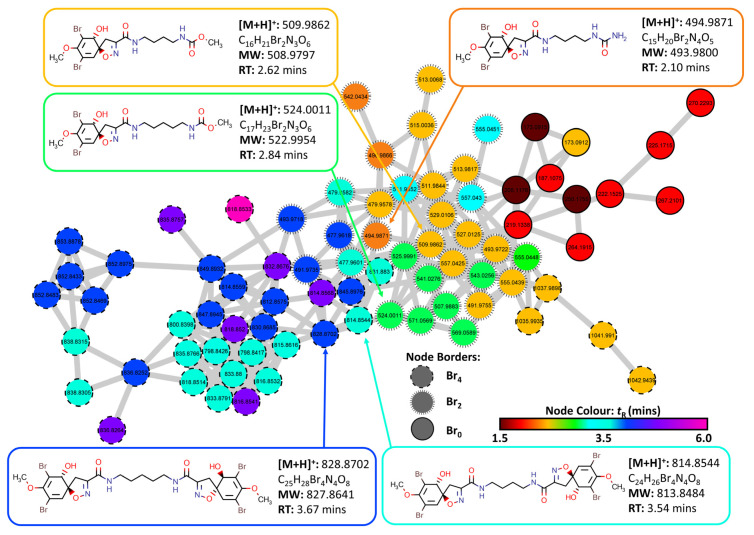
Cluster 6 containing spiroisoxazoline compounds and aerothionin derivatives extracted from the FBMN created from MS/MS data collected from analysis of the crude extracts of the four target organisms (2018_59, 2018_61, 2018_62 and 2018_63). The Node Border displays the number of bromine atoms present as indicated by MS1 data. Node color displays the retention time of each feature to identify potential adducts and different isotopes for individual compounds.

Compounds from the candidate target list were then manually searched within the network using the MS1 parent mass as a prerequisite for identification, followed by manual MS2 inspection where a further 33 compounds were identified as putative annotations (Supplementary Materials Table S2 [10,27,28,33,66,69,70,71,75,87,88,89,90,91,92,93,94,95,96,97,98,99]). Cluster 6 contained a set of nodes that matched potential MS molecular ion features of spiroisoxazolines within the target list, Figure 5. Retention times as well as prospective number of bromine atoms was used to assist in annotation. As this cluster contained no ISDB annotations or GNPS library hits, literature reference data was used to help identify the compounds present within this cluster [73,74,77,87]. Initially, the compound aerothionin (**2**) annotated the node with parent mass *m/z* 814.8544 [M + H]^+^ (calcd. for [C_24_H_26_^79^Br_4_N_4_O_8_ + H]^+^: 814.8562, Δ: −2.2 ppm). Comparison of the MS/MS data with that of the literature showed comparative fragmentation patterns [87]. Four structural derivatives of aerothionin (**2**) were also identified within this cluster including homoaerothionin (**3**) *m/z* 828.8702 [M + H]^+^ (calcd. for [C_25_H_28_^79^Br_4_N_4_O_8_ + H]^+^: 828.8719, Δ: −2.1 ppm), subereamolline C (**12**) *m/z* 509.9862 [M + H]^+^ (calcd. for [C_16_H_21_^79^Br_2_N_3_O_6_ + H]^+^: 509.9875, Δ: −2.5 ppm), subereamolline D *m/z* 524.0011 [M + H]^+^ (calcd. for [C_17_H_23_^79^Br_2_N_3_O_6_ + H]^+^: 524.0032, Δ: −4.0 ppm) as well as a derivative of purealidin L *m/z* 494.9871 [M + H]^+^ (calcd. for [C_15_H_20_^79^Br_2_N_4_O_5_ + H]^+^: 494.9879, Δ: −1.8 ppm) which contained a urea derived end group functionality rather than the more frequently observed guanidine moiety.

**Figure 6:**
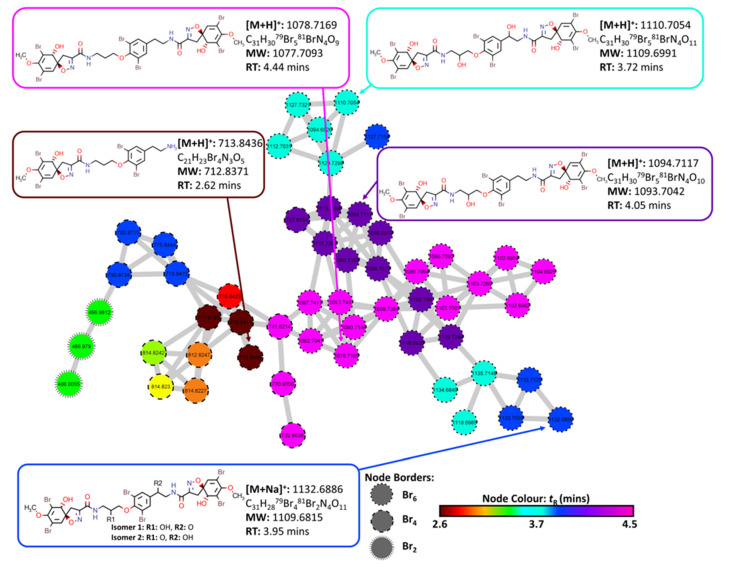
Cluster 13 containing bromotyramine linked spiroisoxazoline compounds and aerothionin derivatives extracted from the FBMN created from MS/MS data collected from analysis of the crude extracts of the four target organisms (2018_59, 2018_61, 2018_62 and 2018_63). Node border displays the number of bromine atoms present as indicated by MS1 data. Node color displays the retention time of each feature to identify potential adducts and different isotopes for individual compounds.

A second cluster was identified which also contained nodes that pertain to spiroisoxazoline compounds that were present within the prospective targets list, Figure 6. However, these were identified to be fistularin-3 derivatives, biosynthetically derived from the addition of spiroisoxazoline head groups to the body of bromotyramine units such as moloka’iamine. This biosynthetic insight proved useful in the dereplication of these compounds via manual inspection of MS2 data. The compound 11,17-deoxyfistularin-3 (**5**) *m/z* 1078.7169 [M + H]^+^ (calcd. for [C_31_H_30_^79^Br_5_^81^BrN_4_O_9_ + H]^+^: 1078.7171, Δ: −0.2 ppm) was annotated upon inspection of the MS2 data after the node had matched compound **5** in the target hit list using MS1 values. The MS2 data contained diagnostic fragment ions consolidating the putative annotation of this compound. Several derivatives were also identified from this cluster including fistularin-3 *m/z* 1110.7054 [M + H]^+^ (calcd. for [C_31_H_30_^79^Br_5_^81^BrN_4_O_11_ + H]^+^: 1110.7070, Δ: −1.4 ppm), 17-deoxyfistularin-3 (**4**) *m/z* 1094.7117 [M + H]^+^ (calcd. for [C_31_H_30_^79^Br_5_^81^BrN_4_O_10_ + H]^+^: 1094.7120, Δ: −0.3 ppm), 11,17-deoxyfistularin-3 (**5**) *m/z* 1078.7169 [M + H]^+^ (calcd. for [C_31_H_30_^79^Br_5_^81^BrN_4_O_9_ + H]^+^: 1078.7171, Δ: −0.2 ppm) and the biosynthetic precursor to fistularin-3, araplysillin I (**11**) *m/z* 713.8436 [M + H]^+^ (calcd. for [C_21_H_23_Br_4_N_3_O_5_ + H]^+^: 713.8449, Δ: −1.8 ppm).

**Figure 7:**
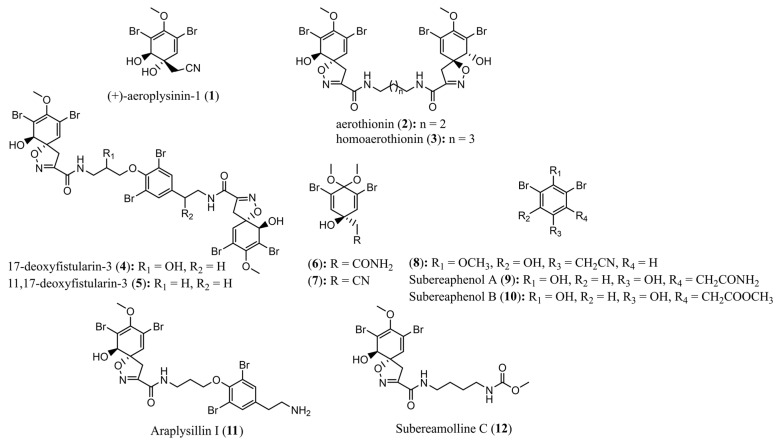
Structures of compounds **1**−**12** isolated from the marine sponge *Pseudoceratina durissima*.

All compounds contained within these clusters were present in the MeOH extract of the marine sponge specimen *Pseudoceratina durissima* 2018_62 as well as small amounts within the DCM extract of the same sponge specimen. The other three sponge specimens (2018_59, 2018_61 and 2018_63) showed low proclivity towards the biosynthesis of spiroisoxazoline compounds, making them less desirable targets for isolation. This, combined with the total number of successfully annotated compounds that existed within the target list, provided sufficient impetus to isolate and test compounds derived from the marine sponge specimen 2018_62. Using this as a dereplication strategy, a total of 12 previously reported secondary metabolites (**1**–**12**) were isolated from the sponge specimen *Pseudoceratina durissima* 2018_62 and confirmed by comparison of their spectral data to literature references, Figure 7.

Analysis of the crude methanolic extract of the sponge sample 2018_62 using analytical HPLC resulted in the observation of two types of key UV chromophore (λ_max_: 280−290 nm and λ_max_: 290−310 nm). Using the MarinLit database [100] it could be established that these chromophores represent two varieties of chemotypes. This included compounds that incorporated scaffolds of spiroisoxazolines, bromotyramines or dibromocyclohexadiene that exhibit chromophores between λ_max_: 280−290 nm [33,69,71,83,94,101,102,103,104,105,106] and compounds of smaller molecular weight containing a phenolic moiety such as verongiabenzenoids and verongiaquinols exhibit chromophores between λ_max_: 290−310 nm [27,32,107,108,109,110].

Combining the observed chromophores with the evidence of highly brominated compound classes, as deduced by the MN study, allowed for the tentative conclusion that the major chemotype present in this extract was the bromotyrosine derived alkaloids. Thus, the crude methanolic extract of the sponge specimen 2018_62 was prioritized for isolation and separated via C_18_ Vacuum Liquid Chromatography (VLC) to yield 20 fractions. Fraction 10 was further purified using RP-HPLC targeting chromatographic peaks of interest that had matching putative annotations to compounds within the target as well as UV chromophores that could potentially be assigned to the identified chemotypes. This resulted in the isolation of the compounds (+)-aeroplysinin-1 (**1**), aerothionin (**2**), subereaphenol B (**10**) and subereamolline (**12**). Fraction 12 was then subjected to Sephadex LH-20 column chromatography yielding a total of 35 fractions. Of these, fractions 21–30 were analyzed using analytical HPLC and several characteristic UV maxima were observed within the range 280−290 nm suggesting the presence of the spiroisoxazoline chemotype. Four compounds, aerothionin (**2**), homoaerothionin (**3**), 17-deoxyfistularin-3 (**4**) and 11,17-deoxyfistularin-3 (**5**), were subsequently isolated from this mixture via C_18_ reversed phased semi-preparative RP-HPLC. Fractions 7 and 8 from the VLC column were then combined and subjected to Sephadex LH-20 column chromatography. This yielded a further 30 fractions of which fractions 17−20 displayed UV chromophores between 280−290 nm and between 290−310 nm, characteristic of a variety of chemotypes. Subsequent purification via C_18_ semi preparative RP-HPLC resulted in the isolation of (+)-aeroplysinin-1 (**1**), 2-(3,5-dibromo-1-hydroxy-4,4-dimethoxycyclohexa-2,5-dien-1-yl)acetamide (**6**), subereaphenol A (**9**) and araplysillin I (**11**).

The DCM crude extract of this sponge specimen was subjected to silica gel flash column chromatography and yielded 17 fractions. Fraction 8 was further purified using C_18_ reversed phase semi-preparative HPLC resulting in the isolation of 2-(3,5-dibromo-1-hydroxy-4,4-dimethoxycyclohexa-2,5-dien-1-yl)acetamide (**6**), (+)-aeroplysinin-1 (**1**), 2-(3,5-dibromo-1-hydroxy-4,4-dimethoxycyclohexa-2,5-dien-1-yl)acetonitrile (**7**) and 2-(3,5-dibromo-2-hydroxy-4-methoxyphenyl)acetonitrile (**8**). All isolated compounds were characterized via 1D and 2D NMR and low-resolution mass spectrometry which was then compared to the high-resolution mass spectrometry data obtained of the crude extracts. All structures were also confirmed by comparison with literature references [27,28,29,30,31,32,33,34].

### 2.4. Bioactivity Testing of Extracts and Compounds

NPs isolated from the marine sponge *Pseudoceratina durissima* (2018_62) were subjected to antimicrobial and anthelmintic as well as zebrafish behavioral toxicity assays to assess the potential neurological interaction that these compounds may possess. All compounds tested were of purity >95% as determined by ^1^H NMR.

#### 2.4.1. Antimicrobial Assay Results

All compounds tested were shown to have minimal cytotoxicity against human embryonic kidney cells (CC_50_: >32 µg/mL) as well as low lysis potential when tested for haemolysis (CC_10_: >32 µg/mL), Table 3, apart from suberaphenol A (**9**) which showed a potent ability to lyse human red blood cells (CC_10_: 1.032 µg/mL). The significantly lower haemolysis activity in the structure derivative subereaphenol B (**10**) suggests that the amide functional group of subereaphenol A (**9**) plays a major role in the lysis activity.

Compounds **1**−**3** and **5**−**12** were assayed against a selection of medically important pathogenic microorganisms (MRSA, *E. coli*, *K. pneumoniae*, *A. baumanii*, *P. aeruginosa*, *C. albicans*, *C. neoformans*), Table 4. Compounds **2**−**3** and **5**−**12** displayed MIC values greater than 32 µg/mL against all organisms. Whereas the compound (+)-aeroplysinin-1 (**1**) showed an MIC value of 32 µg/mL displaying a mean value of 96.3% inhibition against MRSA and 94.5% inhibition against *E. coli*. The MIC was determined to be between the concentration range of 0.25−32 µg/mL and defined as the concentration at which growth was inhibited by ≥80%. Interestingly, this compound has previously displayed antibacterial activity to the non-multidrug resistant strain of *Staphylococcus aureus* [15,111]. To the best of our knowledge this compound had not been tested against any drug resistant strain of Gram-positive bacterium until this study. Previously, (+)-aeroplysin-1 (**1**) has exhibited an MIC of 25 µg/mL against *S. aureus* ATCC 25923 as well as large inhibition zones being observed when tested at 100 µg/mL in disc diffusion assays against *S. aureus* [15].

The marine compound (+)-aeroplysin-1 (**1**) has also been reported to display significant activity against HIV with an IC_50_ of 14.6 µM [112] but to date most of the work on this compound as a potential pathogenic agent has been overshadowed by significant developments in the understanding of this compound’s potential as an anticancer agent [113,114,115,116]. The potent activity of (+)-aeroplysinin-1 (**1**) observed against MRSA in this current work warrants further investigation to evaluate the potential of (+)-aeroplysinin-1 (**1**) as an antibiotic against multidrug resistant pathogens.

#### 2.4.2. Anthelmintic Assay Results

NPs isolated with sufficient mass (**2**–**4** and **6**–**8**) were tested for anthelmintic activity against exsheathed third-stage larvae (xL3s) of the blood-feeding parasitic nematode *Haemonchus contortus*. NPs (**2**–**4** and **6**–**8**) were tested on the xL3s in dose–response evaluations to establish whether any of these compounds inhibited larval motility at 72 h and/or development after 7 days of compound exposure. None of these compounds significantly affected larval motility or development.

#### 2.4.3. Zebrafish Assay Results

Behavioral endpoints in zebrafish assays (photo motor response and locomotor response) have become popular predictors for the mechanism of action of prospective neurologically active drugs [117,118,119]. High-throughput models using the zebrafish (*Danio rerio*) have been established and are becoming more commonplace in early drug discovery campaigns to investigate potential neurotoxic side effects of drugs [119,120].

Bromotyrosine derived alkaloids from the Verongiida marine sponges have long been implicated as a feeding deterrent for potential predatory fish species. Dosed feeding experiments using both the crude extracts and the brominated tyrosine derivative psammaplysene D isolated from the Verongiida sponge *Suberea ianthelliformis* showed significant curtailing in feeding behavior of both the reef fish (*Acanthus triostegus*) and fresh water fish (*Poecilia reticulata*) [121]. It was also suggested that the mechanism of this activity could potentially be attributed to the significant neurological interaction and AChE inhibition shown in subsequent assays, citing the common side effect of anorexia due to AChE inhibition treatment as the cause for reduced feeding tendencies. Bromotyrosine alkaloids have previously been shown to exhibit AChE inhibitory qualities [122,123]. Further, it was also noted that during acute exposure to both the crude extract and psammaplysene D treatments (100 µg/mL) the fish became totally confused and experienced uncontrolled mobility in accordance with symptoms of balance loss. Anxiolytic effects were also documented for highly related spiroisoxazolines and bromotyrosine alkaloids isolated from *Aplysina fulva* when observed using an adult zebrafish model system. However, this was attributed to interaction of these compounds with the GABAergic system, where reduced mobility was observed upon treatment, mimicking the behavior of the diazepam positive control [124].

It is possible that this behavior is indicating a neurological interaction of the bromotyrosine compounds with the fish rather than simply being an issue with the palatability of the compound or extract. We propose that bromotyrosine alkaloids produced by the Verongiida marine sponges may produce altered behavior under varying concentrations due to interactions with neurotransmitters. This may be a contributing factor to the documented ecological role of these compounds as a feeding deterrent to predatory fish species.

Crude extracts and pure compounds isolated from the Verongiida marine sponges, *Aplysina aerophoba* and *Aplysina cavernicola*, were also reported as exhibiting feeding deterrent behavior [13]. The compounds aerothionin and aplysinamisin-1 were both shown to exhibit significant feeding deterrent activity when offered to the Mediterranean fish species *Blennius sphinx*. Interestingly, a specialized predator of Verongiida marine sponges, the opisthobranch (*Tylodina coricalis*), has also been reported to sequester bromotyrosine derived alkaloids in order for it to also avoid predation [125].

Despite bromotyrosine derived metabolites having thus far been clearly shown to exhibit a physiological feeding deterrent effect on marine predators, to date there has been little investigation into the anxiolytic effects induced by these compounds. This information is necessary in both the development of these compounds as leads and may also play a role in the understanding of the ecological function they play for the Verongiida sponges.

**Figure 8:**
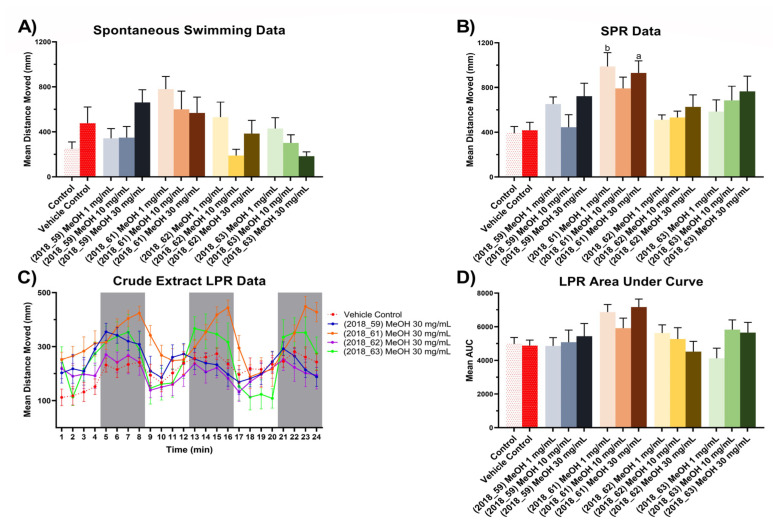
Zebrafish assay results for crude extracts (2018_59, 2018_61, 2018_62 and 2018_63). (**A**) Spontaneous, unstimulated swimming bioassay results for crude extracts (2018_59, 2018_61, 2018_62 and 2018_63) at concentrations of 1 mg/mL, 10 mg/mL and 30 mg/mL, (**B**) Stimulated Predator Response (SPR) bioassay results for crude extracts (2018_59, 2018_61, 2018_62 and 2018_63) at concentrations of 1 mg/mL, 10 mg/mL and 30 mg/mL, (**C**) Larval Photo-motor Response (LPR) bioassay results for crude extracts (2018_59, 2018_61, 2018_62 and 2018_63) at 30 mg/mL. All results for (**A**–**C**) are shown as mean ± SEM, *n* = 9. Results for (**D**) are shown as mean of the area under the curve ± SEM, *n* = 9. Significant difference between treatment and vehicle control indicated by letter a, b, c or d. (one-way ANOVA, Tukeys test, a: *p* < 0.05, b: *p* < 0.01, c: *p* < 0.001, d: *p* < 0.0001). Where no significant difference was observed between treatment and vehicle control no letter was included.

For the zebrafish bioactivity assay, three experiments were performed on 5 days post fertilization (dpf) zebrafish larvae treated with methanol crude extracts from all four sponge specimens, using total distance moved as an endpoint metric. Spontaneous swimming experiments were performed according to the following protocol: allowing a 2 min acclimatization period in darkness followed by a 5 min darkness swimming period, where total distance moved was measured. Simulated predator response (SPR) experiments were performed involving a 2 min acclimatization period in light conditions, followed by 3 min in light free swimming and a measurement period of 3 mins in dark. Finally, a Larval Photo-motor Response (LPR) experiment was conducted beginning with 2 mins light acclimatization followed by three cycles of alternating photic stimulus (each cycle consisting of 4 min light –4 min dark conditions to assess the profile of the treatment across time, Figure 8.

The results presented in Figure 8A show that MeOH extracts from 2018_61 and 2018_62 appeared to induce dose dependent hyperactivity at lower concentrations compared to the vehicle control for the spontaneous swimming experiment with a reduction of mean total distance moved when tested with higher concentrations. The MeOH extract of 2018_63 induced hypoactivity at low concentrations, but at higher concentrations it followed the same trend as 2018_61, with a reduction in mean total distance moved. However, these results were not deemed significantly different than the vehicle control upon analysis with one-way ANOVA and Tukey’s mean comparison test. In the SPR experiment, the 2018_61 extract maintained a hyperactivity effect at the lowest concentration (one-way ANOVA, *p* < 0.01) but this effect dipped showing monophasic suppression of hyperactivity in the 10 mg/mL treatment, Figure 8B. Whilst no significant difference was observed in the SPR data relative to vehicle control for extracts 2018_62 and 2018_63 it is worth noting that both extracts followed a pattern of monotonic stimulation.

LPR experiments revealed the extract from 2018_61 to exhibit a hyperactivity effect in both light and dark cycles, whereas the extract from 2018_62 appeared to be the only exposure that induced slight hypoactivity relative to the vehicle control, Figure 8C. To investigate the overall impact of each treatment, the Area Under the Curve (AUC) was calculated for each treatment and represented as mean AUC ± SEM, *n* = 9 (Figure 8D). Whilst no treatment was deemed significantly different to that of the vehicle control effects, it is interesting to note that the extract from 2018_62 illustrated monotonic suppression with increasing concentration of the extract. This type of effect was deemed noteworthy as it reflects that of AChE inhibition shown by treatment of larvae with organophosphates, where low concentrations result in hyperactivity due to the higher likelihood of an action potential occurring when acetylcholine gated ion channels are opened. However, higher concentrations of organophosphates usually result in hypoactivity and eventually paralysis due to over excitation [117]. The decreasing hyperactivity response of zebrafish larvae treated with the crude extract 2018_62 during LPR experiments highlights the potential for isolation of antimicrobial agents that will possibly have minimal undesired interactions with the central nervous system.

**Figure 9:**
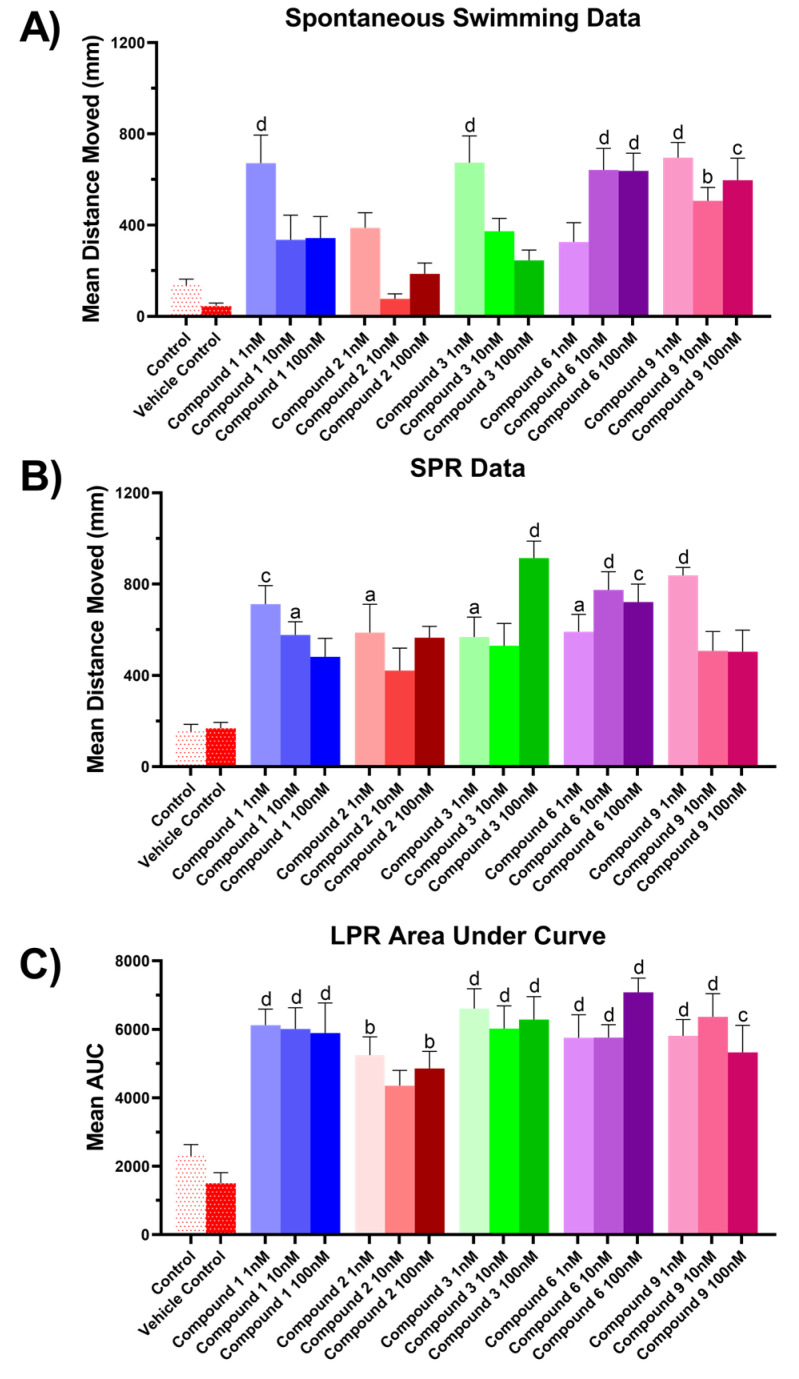
Zebrafish assay results for compounds **1**–**3**, **6** and **9**. (**A**) Spontaneous, unstimulated swimming bioassay, (**B**) SPR bioassay, (**C**) LPR bioassay. All results for (**A**) and (**B**) are shown as mean ± SEM, *n* = 9. Results for (**C**) are shown as mean of the area under the curve ± SEM, *n* = 9. Significant difference between treatment and vehicle control indicated by letter a, b, c or d. (one-way ANOVA, Tukeys test, a: *p* < 0.05, b: *p* < 0.01, c: *p* < 0.001, d: *p* < 0.0001). Where no significant difference was observed between treatment and vehicle control no letter was included.

Subsequently, compounds **1–3**, **6** and **9** isolated from extract 2018_62 were tested under the same experimental conditions as the crude extracts. Previous zebrafish assays on structurally related bromotyrosine derivatives containing the same spiroisoxazoline functionality explored toxicity-based mortality in zebrafish larvae at 5 dpf, and observed significant larval mortality for aerophobin-1 and aerophobin-2 only above concentrations of 1 µM [126]. Thus, to maintain fish activity so that a behavioral endpoint could be observed in these experiments, pure compounds were tested at concentrations of 1 nM, 10 nM and 100 nM to prevent mortality. Spontaneous, unstimulated swimming data illustrated significant hyperactivity for the 1 nM treatments of (+)-aeroplysinin-1 (**1**) (*p* < 0.0001), homoaerothionin (**3**) (*p* < 0.0001) and subereaphenol A (**9**) (*p* < 0.0001) all of which showed reduced mean distance moved for the lower concentrations of 10 nM and 100 nM, Figure 9A. Aerothionin (**2**) showed no significant difference to the vehicle control for all three concentrations while 2-(3,5-dibromo-1-hydroxy-4,4-dimethoxycyclohexa-2,5-dien-1-yl)acetamide (**6**) displayed hyperactivity at 10 nM (*p* < 0.0001) but was curtailed with the 10-fold increase in treatment concentration at 100 nM (*p* < 0.0001).

Homoaerothionin (**3**) displayed an interesting dose dependent hypoactivity effect during the free-swimming experiment which was the opposite to what was observed in the SPR experiment. Clear hyperactivity was observed for this compound at the higher dosage of 100 nM (*p* < 0.0001) indicating a significant neurotoxicological interaction increasing anxiety-like behavior during the simulated predator response experiment, Figure 9B. This is interesting given the structural similarities of aerothionin (**2**) and homoaerothinin (**3**) indicating the length of the central lipidic chain of (CH_2_)_5_ for homoaerothionin (**3**) and (CH_2_)_4_ for aerothionin (**2**) as being important for functional modality in anxiolytic response. In contrast to homoaerothinin (**3**), aerothinin (**2**) showed similar effects in all three experiments with a biphasic distribution of mean movement data, potentially illustrating a different mechanism of action with the central nervous system between the two structural derivatives.

(+)-aeroplysinin-1 (**1**) displayed significant hyperactivity effects at the lower concentration of 1 nM (*p* < 0.0001) for both the spontaneous swimming and SPR experiments showing increased hypoactivity with higher concentrations for both experiments illustrating anxiolytic profiles for each, Figure 9A,B.

Photo-motor response results were obtained for all compounds across all three concentrations 1 nM, 10 nM and 100 nM. The mean area under the curve was used to compare compound profiles across light and dark cycles collectively, Figure 9C. This illustrated significant hyperactivities for all compounds at all concentrations except for compound **2**, which was 10 nM (+)-aeroplysinin-1 (**1**) that illustrated reduced hyperactivity at higher concentrations, as was observed in the spontaneous swimming and SPR experiments.

It is possible that exposure to (+)-aeroplysinin-1 (**1**) results in interaction with AChE systems and therefore in more frequent opening of acetylcholine gated sodium channels leading to hyperactivity at low concentrations and over excitation at higher concentrations. However, given the current data this would only be speculative at best. The exposure to the bromotyrosine derived alkaloids was performed over relatively small time periods which may limit the longer term neurobehavioral predictivity of these assays. We suggest that a further step in understanding the interaction of these compounds with the central nervous system would be to quantify the amount of each compound absorbed by the larvae in various body tissues. This would also be more informative if comparisons could be made between total absorption of compounds through both passive feeding as well as acute exposure. In this way it may be possible to distinguish if feeding deterrence occurs through a palatability issue, or through exposure of the central nervous system to these compounds causing neurobehavioral challenges for potential predators to these marine sponges.

### 2.5. Molecular Docking of (+)-Aeroplysinin-1

Molecular docking experiments were performed in silico on the compound (+)-aeroplysinin-1 (**1**) against the crystal structures of 19 prominent targets for the MRSA pathogen, Table 5. In a previous in silico docking study this compound had been shown to bind with high affinity to the target receptor DNA gyrase (GyrB) and this was postulated as the mechanism of action by which (+)-aeroplysinin-1 (**1**) displayed growth inhibitory activity against both *S. aureus* and *E. coli* strains [127]. This, however, was the only target that was modeled against (+)-aeroplysinin-1 (**1**). Our investigations have shown that (+)-aeroplysinin-1 (**1**) has shown high affinity against many potential targets that could be postulated as the mechanistic origin for the inhibitory activity against MRSA. Indeed, this compound showed the strongest binding affinity score with the target protein Thioredoxin reductase (TrxB) which is part of the thioredoxin system responsible for antioxidant protection of the cell through redox regulation achieved by conversion of thiol and disulfide bonds [128]. TrxB has been postulated as a promising target for the development of novel classes of antibiotics against Gram positive bacteria, due in part to the structural differences observed between mammalian and bacterial thioredoxin reductases, as well as its ability to effect mortality in both planktonic and biofilm modes of growth [128,129].

**Figure 10:**
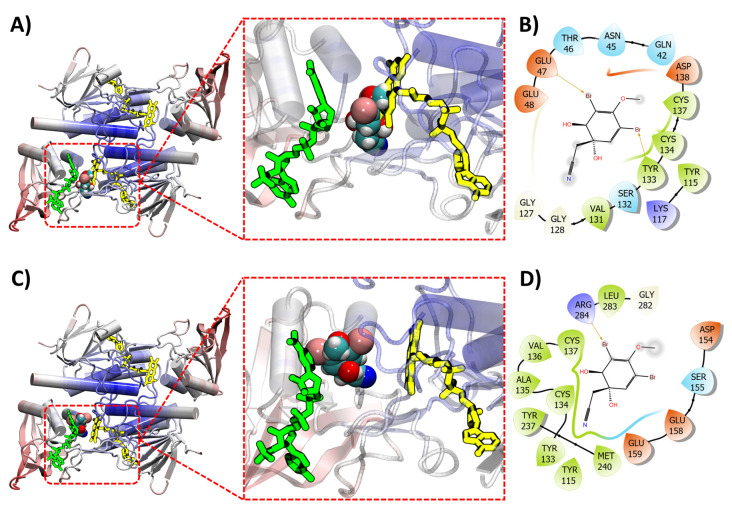
**(A**) (+)-aeroplysin-1 (**1**) (space filling model) shown docking with thioredoxin reductase (TrxB) (ribbon). Docking performed without presence of both FAD (yellow sticks) and NADPH (green sticks). (**B**) Intermolecular interaction between the ligand (+)-aeroplysinin-1 (**1**) and the target thioredoxin reductase (no FAD or NADPH during docking). (**C**) (+)-aeroplysin-1 (**1**) (space filling model) shown docking with thioredoxin reductase (TrxB) (ribbon). Docking performed with FAD (yellow sticks) present but not NADPH (green sticks). (**D**) Intermolecular interaction between the ligand (+)-aeroplysinin-1 (**1**) and the target thioredoxin reductase (FAD present during docking).

Thioredoxin reductase exists with a Flavin Adenine Dinucleotide (FAD) cofactor which plays a crucial role, together with Nicotinamide Adenine Dinucleotide Phosphate (NADPH), in the reduction of thioredoxin, ultimately helping to maintain a homeostatic redox equilibrium and protect the cell from oxidative damage [130]. FAD accepts reducing equivalents from NADPH and transfers them to a dual cysteine functionality adjacent to the isoalloxazine ring of FAD represented by the series of residues CxxC (Cys134, xx, Cys137) within the *S. aureus* TrxB (4gcm) [131,132]. This particular set of residues are part of the active binding pocket for thioredoxin and offer a good target for potential inhibitors [133].

(+)-aeroplysinin-1 (**1**) binds with the TrxB receptor adjacent to the FAD pocket within the region of the CxxC functionality, sharing common residues with the isoalloxazine ring of FAD, Figure 10A. Figure 10A depicts the overlaying of FAD in its normal binding position after (+)-aeroplysinin-1 (**1**) has been docked with TrxB where FAD can be seen overlapping the ligand. This appears to occur when docking is performed both with the presence of the FAD cofactor as well as without, Figure 10C. A preference is observed for (+)-aeroplysinin-1 (**1**) to interact with residues that would otherwise be occupied by the isoalloxazine ring of FAD when docking is performed without the presence of FAD. However, when the cofactor is included, (+)-aeroplysinin-1 (**1**) still appears to bind within the active pocket interacting with the CxxC motif, with both Cys134 and Cys137 forming close contacts with the ligand for both FAD-bound and FAD-free TrxB, albeit in a slightly altered position and conformation, Figure 10B,D. This suggests that this compound could potentially block the interaction of NADPH with the TrxB system, ultimately inhibiting the interaction of FAD and NADPH during the thioredoxin reductase cycle. However, further experimental investigations would be necessary to confirm this theory.

### 2.6. Limitations and Perspectives

The virtual screening strategy employed in this work has led to the isolation of the compound (+)-aeroplysinin-1 (**1**). Many of the other candidate compounds isolated exhibited minimal activity when tested against MRSA at comparable concentrations. This result highlights limitations in the screening strategy employed that contribute to the isolation of false positives. We propose that this can be attributed to three main reasons; i) The assumption of similarity and activity, ii) Selection of target enzymes may not be optimal for the database of compounds, and iii) Predictive limitations in both docking calculations and reporting of prior actives in databases. The assumption of similarity and activity is a persistent theme in the use of any similarity metric to select drug targets. This assumption inherently ignores the existence of so called ‘activity cliffs’ when looking at structurally similar compounds. It may be the case that actives were isolated but there was a large difference in potency of these compounds and thus at the concentration tested they were deemed to be inactive. False positive selection by the virtual screening process is indicative of a process that requires further optimization. This could be achieved by utilizing a micro fractionation bioassay guided approach comparing activity of fractions containing predicted actives against those that do not.

Target enzyme selection also plays a major role in virtual screening strategies especially those that judge success based on whole organism assay outcome. In the current work the aim was to select a large array of the most promising targets currently available in the literature. However, this leaves the potential for false negatives since target selection can inevitably exclude active compounds that may be active via a mechanism of action that is not under investigation in the screening process. Further, the docking calculations provide a representation of interaction affinity between a ligand and a target enzyme, and this does not translate directly to growth inhibition of that compound when assayed against a whole organism. This, combined with the potential for prior actives to be reported at varying concentrations under different conditions, introduces some limitations in the strategy used here as compared to a model-based strategy. Further, it should be noted that virtual screening based on cluster populations results in the exclusion of compounds that are singletons. This was deemed necessary as some compounds in the data set can have such a large degree of structural dissimilarity to others that fair comparisons between molecular docking affinities cannot be made. Thus, this strategy is best optimized for a data set that is already either of a single structure class of ligands or a set of structure classes that are highly similar.

Nevertheless, this strategy has been shown to be effective for the quick identification of targets when a model-based strategy is not an option due to lower quality literature data. Future optimization of this strategy will involve investigation and implementation of a standardized activity score for different methods of whole organism assays. Efforts in the future could attempt to implement micro fractionation strategies combined with crude extract bioassay in order to assist in better linking the docking predictions with the biological activity.

## 3. Materials and Methods

### 3.1. General Experimental

All organic solvents used were either analytical grade (AR or GR) or HPLC/UV grade. Milli-Q water was obtained from a Millipore Q3 Ultrapure water distillation unit. Optical rotations were obtained using a Rudolph Research Analytical Autopol IV automatic polarimeter equipped with a 1.5 mL cell set to Na 589 nm wavelength. ^1^H (500 MHz), ^13^C (125 MHz), and 1D NOE spectra were acquired in CDCl_3_ or CD_3_OD on a 500 MHz Agilent DD2 NMR spectrometer with referencing to solvents signals (*δ* 7.26 and 77.0 ppm for CDCl3, *δ* 3.31 and 49.0 for CD_3_OD). 2D NMR experiments performed included gCOSY, HSQCAD and gHMBCAD.

Silica gel flash chromatography was carried out using Davisil LC35 A silica gel (40–60 mesh) with a 20% stepwise solvent elution from 100% petroleum ether (60–80 °C) to 100% CH_2_Cl_2_ to 100% EtOAc and finally to 100% MeOH. C_18_ VLC was carried out on silica gel 60 RP-18 (40–63 μm) using a 20% stepwise solvent elution from 100% H_2_O to 100% MeOH, and, finally, to 100% CH_2_Cl_2_.

All analytical reversed phase HPLC analysis and method development was carried out on a Dionex P680 solvent delivery system with a PDA100 UV detector (operating software Dionex Chromeleon, version 6.80, Sunnyvale, CA, USA). All analytical HPLC was performed on an Agilent ZORBAX Eclipse Plus (5 μ), C_18_, 250 × 4.6 mm column using a gradient method (0–2 min 10% CH_3_CN/H_2_O; 14-24 min 75% CH_3_CN/H_2_O; 26–30 min 100% CH_3_CN; and 32–40 min 10% CH_3_CN/H_2_O run at 1.0 mL/min). Semi-Preparative reversed phase chromatography was performed using a Varian Prostar 210 solvent delivery system equipped with a Prostar 335 PDA detector (operated using Varian Star Workstation software, version 6.30, Sunnyvale, CA, USA) using gradient methods. All semi-preparative HPLC was performed on an Agilent ZORBAX Eclipse XDB-C_18_, 250 × 9.4 mm, 5 μm column.

### 3.2. Sponge Material

All specimens of sponge examined in this study were collected via SCUBA just offshore at Queenscliffe, Port Phillip Bay, Victoria, Australia (38˚17.6547’S, 144˚35.8642’E) at a depth of 10–12 m in 2018. Samples were collected in accordance with the Australian Fisheries Act of 1995 under a general research permit granted by Fisheries Victoria (Permit Number: RP717). Sponge taxonomy was assigned by Dr. Lisa Goudie as Demospongiae, Verongiida, Pseudoceratinidae, *Pseudoceratina durissima* Carter, 1885. In total four specimens of sponge were investigated in this study *Pseudoceratina durissima* (2018_59), *Pseudoceratina* cf. *durissima* (2018_61), *Pseudoceratina durissima* (2018_62) and *Pseudoceratina* cf. *durissima* (2018_63). Voucher specimens are deposited in the School of Science, RMIT University (voucher codes: 2018_59, 2018_61, 2018_62, 2018_63) as well as with Museums Victoria (voucher codes: F248107, F248108, F248109, F248110).

### 3.3. Extraction and Isolation

The sponge (179.8 g, wet weight) *Pseudoceratina durissima* (2018_62) was extracted in a solvent system of 3:1 MeOH:DCM (1L). The crude extract was filtered and concentrated using reduced pressure. The crude extract was sequentially solvent partitioned (triturated) into DCM (644.2 mg) and MeOH (5.9 g) soluble extracts, respectively. The DCM extract was subjected to silica gel column chromatography to yield 17 fractions (20% stepwise elution from petroleum ether (60–80 ˚C) to DCM to EtOAc and, finally, to MeOH). The 20% DCM/EtOAc fraction was subjected to semi-preparative RP-HPLC (50% CH_3_CN/H_2_O) to yield aerothionin (**2**) (8.6 mg, 0.03%) and homoaerothionin (**3**) (4.9 mg, 0.02%). The 60% DCM/EtOAc fraction was subjected to semi-preparative RP-HPLC (using a gradient of 0–2 mins 35% CH_3_CN/H_2_O; 25–30 mins 75% CH_3_CN/H_2_O; 34-38 mins 100% CH_3_CN; and 39–40 mins 10% CH_3_CN/H_2_O) to yield 2-(3,5-dibromo-1-hydroxy-4,4-dimethoxycyclohexa-2,5-dien-1-yl)acetamide (**6**) (0.9 mg, 0.003%), (+)-aeroplysinin-1 (**1**) (2.9 mg, 0.01%), 2-(3,5-dibromo-1-hydroxy-4,4-dimethoxycyclohexa-2,5-dien-1-yl)acetonitrile (**7**) (1.6 mg, 0.006%) and 2-(3,5-dibromo-2-hydroxy-4-methoxyphenyl)acetonitrile (**8**) (0.7 mg, 0.003%).

The MeOH extract was subject to C_18_ VLC (20% stepwise elution from H_2_O to MeOH and then to DCM and finally flushed using 0.1% trifluoroacetic acid (TFA) in MeOH) resulting in 20 fractions. The 20% H_2_O/MeOH fraction was then purified using semi-preparative RP-HPLC (using a gradient of 0 mins 10% CH_3_CN/H_2_O; 70 mins 60% CH_3_CN/H_2_O; 75–80 min 10% CH_3_CN/H_2_O) to yield (+)-aeroplysinin-1 (**1**) (2.0 mg, 0.007%), aerothionin (**2**) (0.8 mg. 0.003%), subereaphenol B (**10**) (1.0 mg, 0.004%) and subereamolline C (**12**) (1.0 mg, 0.004%).

The MeOH fraction was then subjected to Sephadex LH-20 column chromatography (100% MeOH) yielding a further 35 fractions. Fractions 21–30 were then mixed and purified using semi-preparative RP-HPLC (using a gradient of 0 min 25% CH_3_CN/H_2_O; 100 min 70% CH_3_CN/H_2_O) to yield aerothionin (**2**) (11.8 mg, 0.04%), homoaerothionin (**3**) (4.0 mg, 0.01%), 17-deoxyfistularin-3 (**4**) (1.3 mg, 0.005%) and 11,17-deoxyfistularin-3 (**5**) (2.1 mg, 0.008%). The 60% H_2_O and 40% H_2_O fractions were then mixed and subjected to Sephadex LH-20 column chromatography (100% MeOH) yielding 30 fractions. Fractions 17–20 were mixed and purified using semi-preparative RP-HPLC (using a gradient of 0–8 min 10% CH_3_CN/H_2_O; 70 min 60% CH_3_CN/H_2_O; 75–80 min 10% CH_3_CN/H_2_O) yielding the compounds (+)-aeroplysinin-1 (**1**) (2.3 mg, 0.009%), 2-(3,5-dibromo-1-hydroxy-4,4-dimethoxycyclohexa-2,5-dien-1-yl)acetamide (**6**) (0.7 mg, 0.003%), subereaphenol A (**9**) (6.9 mg, 0.03%) and araplysillin I (**11**) (1.3 mg, 0.005%). All percentage yields are reported based on the dry mass of the sponge material (27 g).

### 3.4. Compound Data

(+)-aeroplysinin-1 (**1**); isolated as a white amorphous powder; ${\left[\alpha \right]}_{D}^{20}$ +86˚ (c 0.15, MeOH); UV (extracted from PDA) λ_max_: 229, 283 nm; all NMR and MS data were identical to the previously published data [27].

(+)-aerothionin (**2**); isolated as a white amorphous powder; ${\left[\alpha \right]}_{D}^{20}$ +118˚ (c 0.1, MeOH); UV (extracted from PDA) λ_max_: 233, 283 nm; all NMR and MS data were identical to the previously published data [27].

(+)-homoaerothionin (**3**); isolated as a white amorphous powder; ${\left[\alpha \right]}_{D}^{20}$ +61˚ (c 0.2, MeOH); UV (extracted from PDA) λ_max_: 232, 283 nm; all NMR and MS data were identical to the previously published data [27].

(+)-17-deoxyfistularin-3 (**4**); isolated as a white amorphous powder; ${\left[\alpha \right]}_{D}^{20}$ +109˚ (c 0.065, MeOH); UV (extracted from PDA) λ_max_: 282 nm; all NMR and MS data were identical to the previously published data [28].

(+)-11,17-deoxyfistularin-3 (**5**); isolated as a white amorphous powder; ${\left[\alpha \right]}_{D}^{20}$ +134˚ (c 0.1, MeOH); UV (extracted from PDA) λ_max_: 283 nm; all NMR and MS data were identical to the previously published data [28].

2-(3,5-dibromo-1-hydroxy-4,4-dimethoxycyclohexa-2,5-dien-1-yl)acetamide (**6**); isolated as a white amorphous powder; UV (extracted from PDA) λ_max_: 280 nm; all NMR and MS data were identical to the previously published data [29].

2-(3,5-dibromo-1-hydroxy-4,4-dimethoxycyclohexa-2,5-dien-1-yl)acetonitrile (**7**); isolated as a white amorphous powder; UV (extracted from PDA) λ_max_: 274 nm; all NMR and MS data were identical to the previously published data [30].

2-(3,5-dibromo-4-methoxyphenyl)acetonitrile (**8**); isolated as a white amorphous powder; UV (extracted from PDA) λ_max_: 288 nm; all NMR and MS data were identical to the previously published data [31].

Subereaphenol A (**9**); isolated as a white amorphous powder; UV (extracted from PDA) λ_max_: 308 nm; all NMR and MS data were identical to the previously published data [32].

Subereaphenol B (**10**); isolated as a white amorphous powder; UV (extracted from PDA) λ_max_: 301 nm; all NMR and MS data were identical to the previously published data [27].

(+)-Araplysillin I (**11**); isolated as a white amorphous powder; ${\left[\alpha \right]}_{D}^{20}$ +123˚ (c 0.065, MeOH); UV (extracted from PDA) λ_max_: 284 nm; all NMR and MS data were identical to the previously published data [33].

(+)-Subereamolline C (**12**) isolated as a white amorphous powder; ${\left[\alpha \right]}_{D}^{20}$ +104˚ (c 0.05, MeOH); UV (extracted from PDA) λ_max_: 228, 283 nm; all NMR and MS data were identical to the previously published data [34].

### 3.5. Structural Similarity Networking

Structural similarity networks were created representing compounds as nodes and edges linked compounds only if they achieved a Tanimoto score greater than 0.5 as well as having the same Murcko scaffold [134,135] as their respective neighbor. Tanimoto scores were calculated using the Morgan fingerprints (*R* = 2, 2048 bits) performed via the rdkit library in python (ver. 3.10.3). Network visualization and layout was performed using the Gephi networking software package (version 0.9.2) utilizing the Fruchtermann Reingold layout algorithm [136]. Potential target compounds were checked for previous activity against any strain of *S. aureus* using the ChEMBL database accessed using OSIRIS Data Warrior (ver 5.50) and assigned as either active, inactive, or unknown.

### 3.6. Molecular Modeling

#### 3.6.1. Acquisition of Target Compound Structures

Target compounds were acquired from the literature following on from our previous work on the Verongiida marine sponges [9]. Briefly, the literature was surveyed using key word search within the SciFinder database for all species within the order Verongiida. NPs isolated from any species within this order were included in the Verongiida compound database.

#### 3.6.2. Acquisition of Target Protein Structures

The Uniprot data base [137] was used in order to obtain the PDB IDs of the 19 target proteins examined in this study. The structures of the identified proteins were downloaded from the RCSB PDB Protein Data Bank (www.rcsb.org, accessed on: 20 March 2022) in the PDB file format and then examined and compared using the protein visualization software VMD [138]. For proteins from the PDB with missing loop segments, homology modelling was employed using the SWISS-MODEL server [139,140] (www.expasy.org/swissmodel, accessed on: 11 April 2022) to repair the 3D structures of these proteins. Protein structures retrieved from the RCBS database or predicted via homology modelling, were pre-processed using PyRx [141] for subsequent computational docking studies.

#### 3.6.3. Molecular Docking between Compounds and Target Proteins

Computational molecular docking aims to identify the various configurations and orientations of a ligand with respect to a target protein. This results in the identification of possible binding sites on the respective protein as well as an estimate of the overall ligand-binding affinity. Interactions between the Verongiida marine sponges data set and the MRSA target list were predicted using the automated docking software AutoDock Vina (v1.2.0) [142]. All protein and compound files for molecular docking were prepared by the GUI frontend PyRx, which was also employed to produce docking parameter input files. All protein and compound PDBQT files were prepared by PyRx based on their corresponding PDB files. The ‘Maximize’ option in PyRx was used to define the docking boxes around the targets, since it could ensure the availability of the entire protein surface and accessible interior pockets for potential binding of ligands during ‘blind’ docking. A default exhaustiveness value of 8 was set for all molecular dockings. The dockings were performed using facilities hosted at the National Computational Infrastructure Centre (Canberra, Australia) to conduct Autodock Vina calculations.

### 3.7. Data Dependent UHPLC-HRMS/MS Analysis

Chromatographic separation was performed on an Acquity UHPLC system (Waters, Milford, MA, USA) interfaced to a Q-Exactive Plus mass spectrometer (Thermo Scientific, Bremen, Germany), using a heated electrospray ionization (HESI-II) source. The LC conditions were as follows: columns: Waters BEH C18 100 × 2.1 mm, 1.7 μm; mobile phase: (A) water with 0.1% formic acid; (B) acetonitrile with 0.1% formic acid; flow rate: 600 μL/min; injection volume: 1 μL; gradient: linear gradient of 5–100% B over 8 min and isocratic at 100% B for 3 min. In positive ion mode, diisooctyl phthalate C_24_H_38_O_4_ [M + H]^+^ ion (*m/z* 391.28429) was used as internal lock mass. The optimized HESI-II parameters were as follows: source voltage: 3.5 kV (pos), sheath gas flow rate (N_2_): 48 units; auxiliary gas flow rate: 11 units; sparge gas flow rate: 2.0; capillary temperature: 300 °C (pos), S-Lens RF Level: 55. The mass analyzer was calibrated using a mixture of caffeine, methionine-arginine-phenylalanine-alanine-acetate (MRFA), sodium dodecyl sulfate, sodium taurocholate and Ultramark 1621 in an acetonitrile/methanol/water solution containing 1% formic acid by direct injection. The data-dependent MS/MS events were performed on the four most intense ions detected in full scan MS (Top4 experiment). The MS/MS isolation window width was 2 Da, and the normalized collision energy (NCE) was set to 35 units. In data-dependent MS/MS experiments, full scans acquired at a resolution of 35,000 FWHM (at *m/z* 200) and MS/MS scans at 17 500 FWHM both with a maximum injection time of 50 ms. After being acquired in the MS/MS scans, parent ions were placed in a dynamic exclusion list for 3.0 s.

### 3.8. Molecular Network Analysis

The MS data were converted from a .RAW (Thermo) standard data file format to .mzXML format using the MSConvert software, part of the ProteoWizard package. The converted files were analyzed using the MzMine software suite v. 2.38. The parameters were adjusted as follows: the centroid mass detector was used for mass detection with the noise level set to 10^6^ for MS level set to 1, and to 0 for MS level set to 2. The ADAP chromatogram builder was used and set to a minimum group size of scans of 5, minimum group intensity threshold of 10^5^, and minimum highest intensity of 10^5^ and *m/z* tolerance of 15.0 ppm. For chromatogram deconvolution, the algorithm used was the wavelets (ADAP). The intensity window S/N was used as S/N estimator with a signal to noise ratio set at 10, a minimum feature height at 500,000, a coefficient area threshold at 130, a peak duration range from 0.02 to 0.5 min and the RT wavelet range from 0.01 to 0.03 min. Isotopes were detected using the isotopes peak grouper with an *m/z* tolerance of 12.0 ppm, an RT tolerance of 0.01 min (absolute), the maximum charge set at 2 and the representative isotope used was the lowest *m/z*. An adduct (Na^+^, K^+^, NH_4_^+^) search was performed at RT tolerance 0.05 min and the maximum relative peak height at 100%. A complex search was also performed using [M + H]^+^ for ESI positive mode, with the RT tolerance set at 0.05 min and the maximum relative peak height at 100%. Peak alignment was performed using the join aligner method (*m/z* tolerance at 15.0 ppm). Eventually, the resulting aligned peak list was filtered using the feature list rows filter option to keep only features associated with MS2 scans.

For the construction of the molecular network, data collected from the MeOH and DCM extracts of all four marine sponge specimens was included (*P. durissima* [2018_59], *P. cf. durissima* [2018_61], *P. durissima* [2018_62] and *P. cf. durissima* [2018_63]).

To keep the retention time, the exact mass information and to allow for the separation of isomers, a feature based molecular network was created using the .mgf file resulting from the MzMine pretreatment step detailed above. Spectral data was uploaded on the GNPS molecular networking platform. A network was then created where edges were filtered to have cosine scores above 0.7 and more than 6 matched peaks. Further edges between two nodes were kept in the network if and only if each of the nodes appeared in each other’s respective top 10 most similar nodes. The spectra in the network were then searched against GNPS’ spectral libraries. All matches kept between network spectra and library spectra were required to have a score above 0.7 and at least 6 matched peaks. The output was visualized using Cytoscape 3.6 software. The GNPS job parameters and resulting data are available at the following address (https://gnps.ucsd.edu/ProteoSAFe/status.jsp?task=9884f3d2122c46bba3ec047adb3ecf3c, accessed on: 19 June 2022). Annotations were achieved using an ISDB strategy previously described [64] together with a taxonomic and cluster oriented strategy [65].

### 3.9. Zebrafish Assays

#### 3.9.1. Zebrafish Larvae Assay Procedures

Basal behavioral analysis was conducted on zebrafish (*Danio rerio*) larvae at 5 dpf using an in-house custom-built behavioral analysis system [143,144]. All assays followed standard protocols as previously described [145,146].

#### 3.9.2. Zebrafish Breeding and Growth

Zebrafish were housed and bred in the AquaCore facility, Monash University, according to standard procedures [147]. Larvae were maintained in E3 media (5mM NaCl, 0.17mM KCl, 0.33 mM CaCl_2_, 0.33 mM MgSO_4_, supplemented with 1 x 10 – 5% methylene blue as antimycotic).

#### 3.9.3. Behavioral Data Analysis

Digital video processing and animal tracking was performed using Ethovision XT ver.16 (Noldus Inc., Wageningen, The Netherlands) using a standardized method as described before [143,144].

#### 3.9.4. Statistical Analysis

All data was averaged across replicate larvae for each treatment and control, respectively, and presented as mean ± SEM. Cumulative distance traveled by larvae was measured by summing the incremental distance moved by each larva between frames of video files. Statistical analysis and data presentation was performed using Prism 8 (GraphPad Software Inc., San Diego, CA, USA). Data sets were tested for outliers and normality of distribution, followed by either one-way or two-way ANOVA testing for significance. If significant exposure effects were displayed, a post hoc Tukey test was then used to compare significance of treatments and vehicle controls. Where time point data was collected significance was compared for each time domain with that of vehicle controls. Significance was universally set to *p* < 0.05.

### 3.10. Antimicrobial Activity Testing

The antimicrobial activity testing was performed by the Community for Antimicrobial Drug Discovery (CO-ADD) [148]. Compounds were assayed against a range of both Gram negative and Gram positive bacteria (see Supplementary Materials S3) including methicillin-resistant *Staphylococcus aureus* (MRSA) (G+), *Escherichia coli* (G-), *Klebsiella pneumoniae* (G-), *Acinetobacter baumannii* (G-), *Pseudomonas aeruginosa* (G-) together with two species of fungus (*Candida albicans* and *Cryptococcus neoformans*).

### 3.11. Anthelmintic Activity Testing

The anthelmintic activity tests were conducted at the University of Melbourne (see Supplementary Materials S4) [149,150]. Dose–response evaluations were carried out to estimate the half-maximal inhibitory concentrations (IC_50_ values) for compounds against xL3s of *H. contortus* in 96-well microtiter plates (Corning, NY, USA) containing 300 xL3s per well. A compound was recorded as having anthelmintic activity if it reduced xL3 motility after 72 h of compound exposure and/or inhibited larval development at 7 days.

## Figures and Tables

**Table 1:** Potential *Staphylococcus* targets list.

Target name	PDBID	MOA	Reference
UDP-N-acetylglucosamine-enolpyruvyl reductase (MurB)	1hsk	Peptidoglycan synthesis	[43]
Isoleucyl-tRNA synthetase	1jzs	Protein modification	[44]
Threonyl-tRNA synthetase	1nyq	Protein modification	[45]
Peptide deformylase (Pdf)	1q1y	Protein modification	[46]
B-ketoacyl-synthase I/II (FabF)	2gqd	Fatty acid synthesis	To be published
D-alanine ligase (Ddl)	2i87	Peptidoglycan synthesis	[47]
Dihydrofolate reductase (DHFR)	2w9h	Cellular regulation	[48]
DNA Gyrase subunit A (GyrA)	2xct	DNA replication	[49]
DNA Gyrase subunit B (GyrB)	3g7b	DNA replication	[50]
Pyruvate Kinase (PK)	3t0t	Glycolysis	[51]
Filamenting Temperature sensitive mutant Z (Ftsz)	3vob	Cell division	[52]
Enoly-acyl-carrier protein reductase (FabI)	4cv1	Fatty acid synthesis	[53]
rRNA methyltransferase	4fak	Protein modification	[54]
Thioredoxin reductase (TrxB)	4gcm	Stress response	To be published
DNA Ligase (LigA)	4glx	Stress response	[55]
DNA Topoisomerase IV subunit B (ParE)	4urn	DNA replication	[56]
Signal Peptidase (SpsB)	4wvj	Protein secretion	[57]
Histidine Kinase (YycG/YycF)	5is1	Cellular regulation	[58]
Peptidoglycan glycosyl transferase (PBP2a)	5m18	Peptidoglycan synthesis	[59]

**Table 2:** Top candidates from the most populated clusters *n* > 12 (where *n* = No. of nodes in cluster).

Structure	Scaffold Class (Cluster)	Cluster Population (*n*)	No. of Lipinski/Veber Failures ^+^	OSIRIS Drug Score ^*^
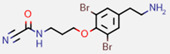	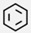	37	0	0.44
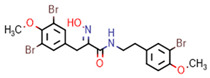	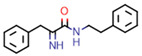	37	1 (MW)	0.48
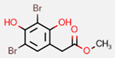	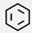	24	0	0.26
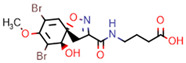	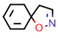	22	0	0.21
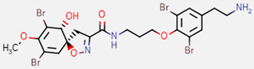	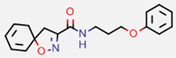	22	1(MW)	0.11
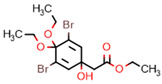	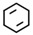	17	0	0.13
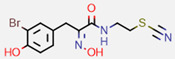	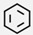	15	0	0.17
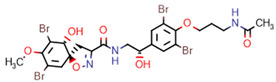	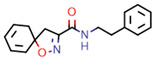	13	1(MW)	0.22
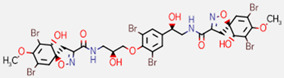	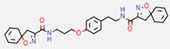	13	5(MW, nHBDon, nHBAcc, nRotB, TPSA)	0.14

* OSIRIS drug score was calculated using the OSIRIS property explorer. It is a value that incorporates the pharmacokinetic properties of each compound together with predicted drug-likeness and the associated toxicity risk for each compound. ^+^ Lipinski’s rules of Drug-likeness: Molecular Weight (MW) < 500Da, LogP_(O/W)_ < 5, No. of Hydrogen Bond Donors (nHBDon) < 5, No. of Hydrogen Bond Acceptors (nHBAcc) < 10. Veber’s rules of oral bioavailability: No. of rotatable bonds (nRotB) < 10, Total Polar Surface Area (TPSA) < 140Å^2^.

**Table 3:** Cytotoxicity and haemolysis assay results for isolated bromotyrosine alkaloids **1**−**3**, **5**−**12**.

Compound	Cytotoxicity ^1^ (CC_50_) µg/mL	Haemolysis ^2^ (HC_10_) µg/mL
**1**	>32	>32
**2**	>32	>32
**3**	>32	>32
**5**	>32	>32
**6**	>32	>32
**7**	>32	>32
**8**	>32	>32
**9**	>32	1.032
**10**	>32	>32
**11**	>32	>32
**12**	>32	>32
**Positive control**	**Tamoxifen:** 9	**Melittin:** 2.7

^1^ Cytotoxicity data was collected by testing compounds against human embryonic kidney cells. ^2^ Haemolysis data was collected by testing compounds against human red blood cells.

**Table 4:** Antibacterial assay results against pathogens for isolated bromotyrosine alkaloids.

Compound	MIC (µg/mL)						
	MRSA ^1^	*E. coli* ^2^	*K. pneumoniae* ^3^	*A. baumannii* ^4^	*P. aeruginosa* ^5^	*C. albicans* ^6^	*C. neoformans* ^7^
**1** **% inhibition**	**32**	**32**	>32	>32	>32	>32	>32
	**95.9, 96.7**	**93.7, 95.2**	−2.8, −3.3	21.5, 52.5	10.9, 18.2	9.5, 9.8	−6.6, 0.0
**2**	>32	>32	>32	>32	>32	>32	>32
**% inhibition**	−1.5, 6.1	0.9, 2.3	10.4, 10.9	−5.2, 11.6	−2.4, 12.1	0.5, 4.2	−9.1, 11.7
**3**	>32	>32	>32	>32	>32	>32	>32
**% inhibition**	−5.9, 7.4	−2.9, −3.1	10.2, 6.6	13.8, 9.8	10.4, 2.2	3.4, 6.3	10.6, 16.1
**5**	>32	>32	>32	>32	>32	>32	>32
**% inhibition**	10.0, 19.8	8.1, 9.5	6.5, 8.7	10.2, 8.5	4.3, 7.2	2.5, 3.3	10.4, 3.8
**6**	>32	>32	>32	>32	>32	>32	>32
**% inhibition**	3.3, 7.0	14.6, 15.6	12.3, 6.4	2.3, 9.7	1.1, 9.7	0.6, 4.6	11.1, 2.0
**7**	>32	>32	>32	>32	>32	>32	>32
**% inhibition**	12.9, 2.5	11.2, 6.2	10.6, 13.3	12.0, 27.4	0.1, 13.2	1.0, 11.2	1.8, 14.1
**8**	>32	>32	>32	>32	>32	>32	>32
**% inhibition**	7.6, 8.5	12.7, 16.9	10.3, 12.5	11.0, 7.5	11.2, 6.8	1.6, 14.3	11.1, 4.0
**9**	>32	>32	>32	>32	>32	>32	>32
**% inhibition**	0.9, 13.3	1.5, 9.6	15.5, 6.6	10.8, 18.5	−1.0, 9.2	−1.2, 12.8	11.2, 11.9
**10**	>32	>32	>32	>32	>32	>32	>32
**% inhibition**	14.9, 4.7	15.8, 6.4	6.1, 9.3	−1.4, −9.2	−4.3, 10.7	−0.8, 13.6	12.4, 8.5
**11**	>32	>32	>32	>32	>32	>32	>32
**% inhibition**	11.3, 5.7	15.9, 8.5	12.2, 17.0	3.4, 7.3	0.3, 8.0	0.6, 16.2	7.6, 7.8
**12**	>32	>32	>32	>32	>32	>32	>32
**% inhibition**	0.4, 8.2	12.3, 6.4	14.0, 5.6	−2.1, 12.7	17.2, 6.3	−3.2, 9.2	−3.4, 8.5
**Positive** **Control**	**Vancomycin:** 1	**Colistin:** 0.125	**Colistin:** 0.25	**Colistin:** 0.25	**Colistin:** 0.25	**Fluconazole:** 0.125	**Fluconazole:** 8

^1^ MRSA (G+) strain ATCC 43300, ^2^ *E. Coli* (G-) strain ATCC 25922, ^3^ *K. pneumoniae* (G-) strain ATCC 700603, ^4^ *A. baumannii* (G-) strain ATCC 19606, ^5^ *P. auruginosa* (G-) strain ATCC 27853, *^6^ C. albicans* (yeast) ATCC 90028, ^7^ *C. neoformans var. grubii* strain ATCC 208821.

**Table 5:** Docking affinity of (+)-aeroplysinin-1 (**1**) against list of potential MRSA targets.

Target Name	PDBID	(+)-Aeroplysinin-1 (1) Affinity (kcal/mol)
Thioredoxin reductase (TrxB)	4gcm	−7.0
Signal Peptidase (SpsB)	4wvj	−6.8
Dihydrofolate reductase (DHFR)	2w9h	−6.7
UDP-N-acetylglucosamine-enolpyruvyl reductase (MurB)	1hsk	−6.5
Peptide deformylase (Pdf)	1q1y	−6.3
Enoly-acyl-carrier protein reductase (FabI)	4cv1	−6.3
Threonyl-tRNA synthetase	1nyq	−6.2
DNA Ligase (LigA)	4glx	−6.2
Isoleucyl-tRNA synthetase	1jzs	−6.1
Filamenting Temperature sensitive mutant Z (Ftsz)	3vob	−5.8
rRNA methyltransferase	4fak	−5.8
DNA Gyrase subunit A (GyrA)	2xct	−5.8
Peptidoglycan glycosyl transferase (PBP2a)	5m18	−5.7
Pyruvate Kinase (PK)	3t0t	−5.6
DNA Topoisomerase IV subunit B (ParE)	4urn	−5.6
B-ketoacyl-synthase I/II (FabF)	2gqd	−5.6
D-alanine ligase (Ddl)	2i87	−5.5
DNA Gyrase subunit B (GyrB)	3g7b	−5.1
Histidine Kinase (YycG/YycF)	5is1	−4.9

## Data Availability

All data are contained within the article and Supplementary Materials.

## Supplementary Materials

The following supporting information can be downloaded at: https://www.mdpi.com/article/10.3390/md20090554/s1, Verongiida data set is included as a .csv file, Table S1: Putative annotations for brominated alkaloids achieved through taxonomy based ISDB comparisons, Table S2: Putative annotations of compounds within hitlist via manual investigation, S3: Antimicrobial activity testing methodology, S4: Anthelmintic activity testing methodology. References [10,17,27,28,33,66,67,68,69,70,71,72,73,74,75,76,77,78,79,80,81,82,83,84,85,86,87,88,89,90,91,92,93,94,95,96,97,98,99,149,150] are cited in the supplementary materials.
