# Supplementary material for: Targeted Isolation of Antibiotic Brominated Alkaloids from the Marine Sponge Pseudoceratina durissima Using Virtual Screening and Molecular Networking
Source: Mar Drugs. 2022 Aug 29;20(9):554. doi: 10.3390/md20090554 (PMC9503778; doi:10.3390/md20090554)
Supplement: Supplementary file 1 [file marinedrugs-20-00554-s001.zip › marinedrugs-1853271 supplementary Materials/SI.pdf]

marine drugs

Targeted Isolation of Antibiotic Brominated Alkaloids from the Marine Sponge *Pseudoceratina durissima* using Virtual Screening and MS/MS Networking

James Lever[1], Florian Kreuder[2], Jason Henry[3], Andrew Hung[1], Pierre-Marie Allard[4], Robert Brkljača[5], Colin Rix[1], Aya C. Taki[6], Robin B. Gasser[6], Jan Kaslin[2], Donald Wlodkowic[3], Jean-Luc Wolfender [7,8] and Sylvia Urban[1,*]

[1] School of Science (Applied Chemistry and Environmental Sciences), RMIT University, GPO Box 2476V Melbourne, VIC 3001, Australia; james.lever@rmit.edu.au (J.L.); colin.rix@rmit.edu.au (C.R.); sylvia.urban@rmit.edu.au (S.U)
[2] Australian Regenerative Medicine Institute, Monash University, Clayton, VIC 3800, Australia; florian.kreuder@monash.edu (F.K), jan.kaslin@monash.edu (J.K)
[3] Neurotoxicology Lab, School of Science (Biosciences), RMIT University, Bundoora, VIC 3083, Australia; s3740720@student.rmit.edu.au (J.H), donald.wlodkowic@rmit.edu.au (D.W)
[4] Department of Biology, University of Fribourg, 1700 Fribourg, Switzerland; pierre-marie.allard@unifr.ch (P.-M.A)
[5] Monash Biomedical Imaging, Monash University, Clayton, VIC 3168, Australia; robert.brkljaca@monash.edu (R.B)
[6] Department of Veterinary Biosciences, Melbourne Veterinary School, Faculty of Veterinary and Agriculture Sciences, The University of Melbourne, Parkville, Victoria, Victoria 3010, Australia; aya.taki@unimelb.edu.au (A.C.T), robinbg@unimelb.edu.au (R.B.G)
[7] School of Pharmaceutical Sciences of Western Switzerland, University of Geneva, CMU, Rue Michel-Servet 1, 1211 Geneve 4, Switzerland; jean-luc.wolfender@unige.ch (J.-L.W)
[8] Institute of Pharmaceutical Sciences of Western Switzerland, University of Geneva, CMU, Geneva, Switzerland; (J.-L.W)
[*] Correspondence: sylvia.urban@rmit.edu.au

Supporting Information:
S1. Brominated alkaloids identified through ISDB annotations
S2. Brominated alkaloids identified through MS1 searching followed by manual MS2 inspection
S3. Antimicrobial activity testing methodology
S4. Anthelmintic activity testing methodology

1. Brominated alkaloids identified through ISDB annotations

Table S1: Putative annotations for brominated alkaloids achieved through taxonomy based ISDB comparisons.

R_t (mins)	*m/z*	Adduct Type	Molecular Formula	Possible Annotation	Cluster number	Reference
2.45	512.0193	$[M+H]^+$	$C_{20}H_{23}{}^{79}Br_2N_3O_3$	Purpuramine D	1	[1]
2.52	526.0332	$[M+H]^+$	$C_{21}H_{25}{}^{79}Br_2N_3O_3$	Purpuramine E	1	[1]
2.78	633.9540	$[M+H]^+$	$C_{22}H_{26}{}^{79}Br_3N_3O_4$	Purpuramine I isomer	1	[1]
1.52	519.0963	$[M+H]^+$	$C_{20}H_{32}{}^{79}Br_2N_4O_2$	Ianthelliformisamine A	2	[2]
1.84	462.0382	$[M+H]^+$	$C_{17}H_{25}{}^{79}Br_2N_3O_2$	Ianthelliformisamine B	2	[2]
2.39	473.9767	$[M+H]^+$	$C_{15}H_{17}{}^{79}Br_2N_5O_3$	Ianthelline	3	[3]
1.16	517.0185	$[M+H]^+$	$C_{17}H_{22}{}^{79}Br_2N_6O_3$	Purealidin A	3	[4]
1.72	489.9719	$[M+H]^+$	$C_{15}H_{17}{}^{79}Br_2N_5O_4$	Purealidin J isomer	3	[5]
2.41	489.9718	$[M+H]^+$	$C_{15}H_{17}{}^{79}Br_2N_5O_4$	Purealidin J isomer	3	[5]
2.51	494.0025	$[M+H]^+$	$C_{15}H_{21}{}^{79}Br_2N_5O_4$	Purealidin L isomer	4	[5, 6]
1.82	494.0026	$[M+H]^+$	$C_{15}H_{21}{}^{79}Br_2N_5O_4$	Purealidin L isomer	4	[5, 6]
1.97	508.0190	$[M+H]^+$	$C_{16}H_{23}{}^{79}Br_2N_5O_4$	Aplysinamisine II	4	[6, 7]
1.85	501.9715	$[M+H]^+$	$C_{16}H_{17}{}^{79}Br_2N_5O_4$	Aplysinamisine I isomer	8	[7]
1.94	501.9715	$[M+H]^+$	$C_{16}H_{17}{}^{79}Br_2N_5O_4$	Aplysinamisine I isomer	8	[7]
1.91	519.9826	$[M+H]^+$	$C_{16}H_{19}{}^{79}Br_2N_5O_5$	14-oxo-aerophobin-2 isomer	8	[8]
1.81	519.9821	$[M+H]^+$	$C_{16}H_{19}{}^{79}Br_2N_5O_5$	14-oxo-aerophobin-2 isomer	8	[8]

2.72	755.8897	$[M+H]^+$	$C_{24}H_{29}{}^{79}Br_4N_3O_5$	Purpurealidin A derivative	10	[9, 10]
2.91	663.9654	$[M+H]^+$	$C_{23}H_{28}{}^{79}Br_3N_3O_5$	Aplysamine -7 isomer	10	[11]
2.54	663.9642	$[M+H]^+$	$C_{23}H_{28}{}^{79}Br_3N_3O_5$	Aplysamine-7 isomer	10	[11]
2.57	540.0486	$[M+H]^+$	$C_{22}H_{27}{}^{79}Br_2N_3O_3$	20-*N*-methylpurpuramine E	10	[12]
2.60	647.9699	$[M+H]^+$	$C_{23}H_{28}{}^{79}Br_3N_3O_4$	Aplysamine-2 isomer	10	[13]
2.84	647.9697	$[M+H]^+$	$C_{23}H_{28}{}^{79}Br_3N_3O_4$	Aplysamine-2 isomer	10	[13]
2.93	741.8746	$[M+H]^+$	$C_{23}H_{27}{}^{79}Br_4N_3O_5$	Purpurealidin J isomer	10	[13]
2.72	741.8748	$[M+H]^+$	$C_{23}H_{27}{}^{79}Br_4N_3O_5$	Purpurealidin J isomer	10	[13]
0.30	393.0165	$[M+H]^+$	$C_{14}H_{22}{}^{79}Br_2N_2O$	Purealidine G isomer	12	[14]
0.70	393.0166	$[M+H]^+$	$C_{14}H_{22}{}^{79}Br_2N_2O$	Purealidine G isomer	12	[14]
2.55	503.9865	$[M+H]^+$	$C_{16}H_{19}{}^{79}Br_2N_5O_4$	Aerophobin-2 isomer	34	[15]
1.86	503.9873	$[M+H]^+$	$C_{16}H_{19}{}^{79}Br_2N_5O_4$	Aerophobin-2 isomer	34	[15]
2.25	421.9703	$[M+H]^+$	$C_{13}H_{17}{}^{79}Br_2N_3O_3$	Moloka'iakitamide	36	[16]
1.63	474.9611	$[M+H]^+$	$C_{15}H_{16}{}^{79}Br_2N_4O_4$	Aerophobin-1	53	[17]
1.84	381.0553	$[M+H]^+$	$C_{15}H_{17}{}^{79}BrN_4O_3$	Verongamine	53	[18]
2.32	458.9658	$[M+H]^+$	$C_{15}H_{16}{}^{79}Br_2N_4O_3$	5-bromoverongamine	53	[19]
1.87	433.9888	$[M+H]^+$	$C_{16}H_{21}{}^{79}Br_2NO_3$	ethyl (Z)-3-(3,5-dibromo-4-(3-(dimethylamino)propoxy)phenyl)acrylate	64	[20]
1.63	466.9740	$[M+H]^+$	$C_{17}H_{16}{}^{79}Br_2N_4O_2$	Ceratamine A	96	[21]
1.71	459.9614	$[M+H]^+$	$C_{14}H_{15}{}^{79}Br_2N_5O_3$	(2Z)-3-(3,5-dibromo-4-hydroxyphenyl)-2-(hydroxyimino)-N-(2-(2-imino-2,3-dihydro-1H-imidazol-4-yl)ethyl)propanimidic acid	301	[22]
0.71	393.0964	$[M+H]^+$	$C_{14}H_{22}{}^{79}Br_2N_2O$	Purealidin G	singleton	[23]

2. Brominated alkaloids identified through MS1 searching followed by manual MS2 inspection

Table S2: Putative annotations of compounds within hitlist via manual investigation.

R_t (mins)	*m/z*	Adduct Type	Molecular Formula	Possible Annotation	Cluster number	Reference
2.62	713.8436	$[M+H]^+$	$C_{21}H_{23}{}^{79}Br_4N_3O_5$	Araplysillin-I	13	[24]
3.54	814.8544	$[M+H]^+$	$C_{24}H_{26}{}^{79}Br_4N_4O_8$	Aerothionin	6	[25]
3.54	831.8830	$[M+NH_4]^+$	$C_{24}H_{26}{}^{79}Br_4N_4O_8$			
3.68	828.8702	$[M+H]^+$	$C_{25}H_{28}{}^{79}Br_4N_4O_8$	Homoaerothionin	6	[26]
3.67	845.8976	$[M+NH_4]^+$	$C_{25}H_{28}{}^{79}Br_4N_4O_8$			
1.97	508.0190	$[M+H]^+$	$C_{16}H_{23}{}^{79}Br_2N_5O_4$	Aplysinamisine II	4	[7]
2.51	494.0025	$[M+H]^+$	$C_{15}H_{21}{}^{79}Br_2N_5O_4$	Purealidin L isomer	4	[5, 6]
1.82	494.0026	$[M+H]^+$	$C_{15}H_{21}{}^{79}Br_2N_5O_4$	Purealidin L isomer	4	[5, 6]
0.57	350.9697	$[M+H]^+$	$C_{11}H_{16}{}^{79}Br_2N_2O$	Moloka'iamine isomer	1	[27]
1.04	350.9694	$[M+H]^+$	$C_{11}H_{16}{}^{79}Br_2N_2O$	Moloka'iamine isomer	1	[27]
3.25	830.8498	$[M+H]^+$	$C_{24}H_{26}{}^{79}Br_4N_4O_9$	11-hydroxyaerothionin	54	[28]
2.93	403.9605	$[M+H]^+$	$C_{13}H_{15}{}^{79}Br_2N_3O_2$	Ceratinamine isomer	194	[27]
3.16	403.9597	$[M+H]^+$	$C_{13}H_{15}{}^{79}Br_2N_3O_2$	Ceratinamine isomer	194	[27]
2.37	337.9022	$[M+H]^+$	$C_9H_9{}^{79}Br_2NO_3$	Aeroplysinin-1 isomer	21	[29]
2.62	466.9443	$[M+H]^+$	$C_{14}H_{16}{}^{79}Br_2N_2O_6$	4-((5S,10R)-7,9-dibromo-10-hydroxy-8-methoxy-1-oxa-2-azaspiro[4.5]deca-2,6,8-triene-3-carboxamido)butanoic acid	105	[30]
1.36	406.9971	$[M+H]^+$	$C_{15}H_{24}{}^{79}Br_2N_2O$	Aplysamine-1	12	[31]

0.66	380.9986	$[M+H]^+$	$C_{13}H_{20}{}^{79}Br^{81}BrN_2O$	3-(4-(2-aminoethyl)-2,6-dibromophenoxy)-N,N-dimethylpropan-1-amine	12	[32]
3.95	693.6981	$[M+H]^+$	$C_{24}H_{28}{}^{79}Br^{81}Br_2N_3O_6$	Purpuramine K	43	[33]
1.11	337.9018	$[M+H]^+$	$C_9H_9{}^{79}Br_2NO_3$	Aeroplysinin-1 isomer	21	[29]
3.72	1112.7031	$[M+H]^+$	$C_{31}H_{30}{}^{79}Br_4{}^{81}Br_2N_4O_{11}$	Fistularin-3	13	[34]
3.18	466.9812	$[M+H]^+$	$C_{14}H_{16}{}^{79}Br_2N_2O_6$	Purpuroacetic acid	13	[35]
4.04	1094.7117	$[M+H]^+$	$C_{31}H_{30}{}^{79}Br_5{}^{81}Br_1N_4O_{10}$	11-Deoxyfistularin-3	13	[36]
2.51	757.8329	$[M+H]^+$	$C_{22}H_{23}{}^{79}Br_4N_3O_7$	Ianthesine B	147	[37]
1.84	462.0382	$[M+H]^+$	$C_{17}H_{25}{}^{79}Br_2N_3O_2$	Ianthelliformisamine B	2	[2]
2.72	619.9381	$[M+H]^+$	$C_{21}H_{24}{}^{79}Br_3N_3O_4$	Purpuramine I isomer	1	[38]
2.84	524.0011	$[M+H]^+$	$C_{17}H_{23}{}^{79}Br_2N_3O_6$	Subereamolline B	6	[39]
2.60	509.9862	$[M+H]^+$	$C_{16}H_{21}{}^{79}Br_2N_3O_6$	Subereamolline A	6	[39]
1.85	501.9715	$[M+H]^+$	$C_{16}H_{17}{}^{79}Br_2N_5O_4$	Aplysinamisine I isomer	8	[7]
1.94	501.9715	$[M+H]^+$	$C_{16}H_{17}{}^{79}Br_2N_5O_4$	Aplysinamisine I isomer	8	[7]
2.91	663.9654	$[M+H]^+$	$C_{23}H_{28}{}^{79}Br_3N_3O_5$	Aplysamine -7 isomer	10	[11]
2.54	663.9642	$[M+H]^+$	$C_{23}H_{28}{}^{79}Br_3N_3O_5$	Aplysamine-7 isomer	10	[11]
2.37	633.9543	$[M+H]^+$	$C_{22}H_{26}{}^{79}Br_3N_3O_4$	Purpuramine H isomer	10	[38]
2.52	633.9545	$[M+H]^+$	$C_{22}H_{26}{}^{79}Br_3N_3O_4$	Purpuramine H isomer	10	[38]
2.45	512.0193	$[M+H]^+$	$C_{20}H_{23}{}^{79}Br_2N_3O_3$	Purpuramine D isomer	1	[1]
2.52	526.0332	$[M+H]^+$	$C_{21}H_{25}{}^{79}Br_2N_3O_3$	Purpuramine E	1	[1]

S3. Antimicrobial activity testing methodology

The compounds submitted to CO-ADD were evaluated in duplicate (n = 2) against seven microorganisms (five bacteria and two fungi) at a concentration of 32 µg/mL in a 384-well, non-binding surface plate (NBS) for each bacterial/fungal strain, keeping the final DMSO concentration to a maximum of 1% DMSO. All bacteria were cultured in cation-adjusted Mueller-Hinton broth (CAMHB) at 37 ˚C overnight. A sample of each culture was then diluted 40-fold in fresh broth and incubated at 37 ˚C for 1.5-3 h. The resultant mid-log phase cultures were diluted (CFU/mL measured by OD_{600}), then added to each well of the compound containing plates, giving a cell density of $5x10^5$ CFU/mL and a total volume of 50 µL. All the plates were covered and incubated at 37 ˚C for 18 h without shaking.

Fungal strains were cultured for 3 days on Yeast Extract-Peptone Dextrose (YPD) agar at 30 ˚C. A yeast suspension of 1 x 10^6 to 5 x 10^6 CFU/mL (as determined by OD_{530}) was prepared from five colonies. The suspension was subsequently diluted and added to each well of the compound-containing plates giving a final cell density of fungi suspension of 2.5 x 10^3 CFU/mL and a total volume of 50 µL. All plates were covered and incubated at 35 ˚C for 24 h without shaking.

Inhibition of bacterial growth was determined measuring absorbance at 600 nm (OD_{600}) using a Tecan M1000 Pro monochromator plate reader. Growth inhibition of *C. albicans* was determined measuring absorbance at 530 nm (OD_{530}), while the growth inhibition of *C. neoformans* was determined measuring the difference in absorbance between 600 and 570 nm ($OD_{600-570}$), after the addition of resazurin (0.001% final concentration) and incubation at 35 ˚C for an additional 2 h. The absorbance was measured using a Biotek Synergy HTX plate reader. The percentage of growth inhibition was calculated for each well, using negative control (medium only) and positive control (bacteria without inhibitors) on the same plate as the references. The significance of inhibition values was determined by modified Z-scores, calculated using the median and MAD of the samples (no controls) on the same plate. Samples with inhibition value above 80% and Z-score above 2.5 for either replicate (n = 2 on different plates) were classed as actives.

Colistin and vancomycin were used as positive bacterial inhibitor standards for Gram-negative and Gram-positive bacteria, respectively. Fluconazole was used as a positive fungal inhibitor standard for *C. albicans* and *C. neoformans*. The antibiotics were provided in four concentrations, with two above and two below its MIC value, and plated into the first eight wells of column 23 of the 384-well NBS plates. The quality control (QC) of the assays was determined by the antimicrobial controls and the Z'-factor (using positive and negative controls). Each plate was deemed to fulfil the quality criteria (pass QC), if the Z'-factor was above 0.4, and the antimicrobial standards showed full range of activity, with full growth inhibition at their highest concentration, and no growth inhibition at their lowest concentration. The seven

test microorganisms were *Staphylococcus aureus* MRSA (ATCC 43300), *Escherichia coli* (ATCC 25922), *Klebsiella pneumoniae* (ATCC 700603), *Acinetobacter baumanii* (ATCC19606), *Pseudomonas aeruginosa* (ATCC 27853), *Candida albicans* (ATCC 90028) and *Cryptococcus neoformans* var. *grubii* (H99; ATCC 208821).

S4. Anthelmintic activity testing methodology

Dose-response evaluations were carried out to estimate the half-maximal inhibitory concentrations (IC_{50} values) for compounds against *Haemonchus contortus* (Haecon-5 strain) exsheathed third-stage larvae (xL3s). *H. contortus* were maintained in experimental sheep and procured in accordance with the institutional animal ethics guidelines and the regulations of Australia (permit no. 1714374; University of Melbourne) [40]. Immediately prior to use, third-stage larvae (L3s) were exsheathed and sterilised by incubation in 0.15% (v/v) bleach at 38 °C for 20 min, followed by washes in sterile saline at room temperature (22-24 °C). After the last wash, xL3s were suspended in sterile lysogeny broth (LB) supplemented with 100 IU/mL of penicillin, 100 µg/mL of streptomycin and 0.25 µg/mL of amphotericin B (Fungizone®, Thermo Fisher Scientific, USA) – designated LB* [40]. Compounds were prepared in two-fold serial dilution, starting at a concentration of 100 µM (18-points; in 50 µl of LB*; 100 µM to 0.76 nM), in 96-well plates (cat. no. 3596; Corning, USA) with larvae dispensed in 50 µL at a density of 300 [40]. LB* + 0.5% DMSO serving as negative control and two reference compounds (20 µM of monepantel (Zolvix, Novartis Animal Health, Switzerland) and 20 µM of moxidectin (Cydectin, Virbac, France)) were applied to the 96-well microtiter plates (Corning, USA) which xL3 (~300/well) were dispensed. Following a 72 h incubation at 38 ˚C and 10% CO_2 with >90% humidity, a video recording (5 sec) was taken of each well of the 96-well microtiter plate containing xL3s using a grayscale camera (Rolera bolt CMOS, Q imaging Scientific, Canada) and a motorized X-Y axis stage (BioPoint 2, Ludl Electronics Products, USA). Individual videos were processed for a motility index (MI) using the unique algorithm written in a custom macro and analyzed through the program ImageJ (v.2.0.0, Fiji) [41]. Seven days after incubation, these compounds were also tested for their ability to inhibit the development of xL3s to L4s by microscopically assessing the development of a mouth and directly compared with negative controls (LB* + 0.5% DMSO). Primary screenings were performed in triplicate, twice, on separate days. A compound was recorded as having activity if it reduced xL3s motility by ≥70% after 72 h of incubation and/or inhibited larval development at 7 days.

References

1. Yagi, H., S. Matsunaga, and N. Fusetani, Purpuramines A-I, New bromotyrosine-derived metabolites from the marine sponge *Psammaplysilla purpurea. Tetrahedron*, **1993**. *49*(18): p. 3749-3754.
2. Xu, M., et al., Ianthelliformisamines A-C, antibacterial bromotyrosine-derived metabolites from the marine sponge *Suberea ianthelliformis. J. Nat. Prod.*, **2012**. *75*(5): p. 1001-1005.
3. Cardenas, P., Who Produces Ianthelline? The Arctic Sponge Stryphnus fortis or its Sponge Epibiont *Hexadella dedritifera*: a Probable Case of Sponge-Sponge Contamination. *J. Chem. Ecol.*, **2016**. *42*(4): p. 339-347.
4. Salim, A.A., Z.G. Khalil, and R.J. Capon, Structural and stereochemical investigations into bromotyrosine-derived metabolites from southern Australian marine sponges, *Pseudoceratina* spp. *Tetrahedron*, **2012**. *68*(47): p. 9802-9807.
5. Kobayashi, J., K. Honma, and M. Tsuda, Purealidins J-R new bromotyrosine alkaloids from the Okinawan marine sponge *Pammplysilla purea. Chem. Pharm. Bull.*, **1995**. *43*(3): p. 403-407.
6. Nicacio, K.J., et al., Cultures of the Marine Bacterium *Pseudovibrio denitrificans* Ab134 Produce Bromotyrosine-Derived Alkaloids Previously Only Isolated from Marine Sponges. *J. Nat. Prod.*, **2017**. *80*(2): p. 235-240.
7. Rodriguez, A.D. and I.C. Pina, The structures of aplysinamisines I, II, and III New bromotyrosine-derived alkaloids from the Caribbean sponge *Aplysina cauliformi. J. Nat. Prod.*, **1993**. *56*(6): p. 907-914.
8. Fendert, T., et al., Bromoisoxazoline alkaloids from the Caribbean sponge *Aplysina insularis. Z. Naturforsch. C*, **1999**. *54*(3-4): p. 246-252.
9. Tilvi, S., et al., New bromotyrosine alkaloids from the marine sponge *Psammaplysilla purpurea. Tetrahedron*, **2004**. *60*(45): p. 10207-10215.
10. Tilvi, S. and M.S. Majik, 2D NMR Studies of Bromotyrosine Alkaloid, Purpurealidin K from Marine Sponge *Psammaplysilla purpurea. ChemistrySelect*, **2019**. *4*(21): p. 6568-6571.
11. Tran, T.D., et al., Bromotyrosine alkaloids from the Australian marine sponge *Pseudoceratina verrucosa. J. Nat. Prod.*, **2013**. *76*(4): p. 516-523.
12. Teruya, T., A. Iwasaki, and K. Suenaga, 20-*N*-methylpurpuramine E: New bromotyrosine-derived metabolite from Okinawan marine sponge *Pseudoceratina purpurea. Bull. Chem. Soc. Jpn.*, **2008**. *81*(8): p. 1026-1027.

13. Tilvi, S. and L. D'Souza, Identifying the related compounds using electrospray ionization tandem mass spectrometry: bromotyrosine alkaloids from marine sponge *Psammaplysilla purpurea. Eur. J. Mass Spectrom. (Chichester)*, **2012**. *18*(3): p. 333-343.
14. Tian, L.W., et al., Aplysinellamides A-C, bromotyrosine-derived metabolites from an Australian *Aplysinella* sp. marine sponge. *J. Nat. Prod.*, **2014**. *77*(5): p. 1210-1214.
15. Weiss, B., et al., Defense metabolites from the marine sponge *Verongia aerophoba. Biochemical Systematics and Ecology*, **1996**. *24*(1): p. 1-12.
16. Badr, J.M., et al., Bioactive brominated metabolites from the Red Sea sponge *Pseudoceratina arabica. J. Nat. Prod.*, **2008**. *71*: p. 1472-1474.
17. Teeyapant, R., et al., Brominated secondary compounds from the marine sponge *Verongia aerophoba* and the sponge feeding gastropod *Tylodina perversa*. *Z. Naturforsch.*, **1993**. *48c*: p. 640-644.
18. Mierzwa, R., et al., Verongamine, a novel bromotyrosine-derived histamine H3-Antagonist from the marine sponge *Verongula gigantea. J. Nat. Prod.*, **1994**. *57*(1): p. 175-177.
19. Thirionet, I., et al., 5-Bromoverongamine, a Novel Antifouling Tyrosine Alkaloid from the Sponge *Pseudoceratina* sp. *Nat. Prod. Lett.*, **1998**. *12*(3): p. 209-214.
20. Kassuhlke, K.E. and J.D. Faulkner, Two new dibromotyrosine derivatives from the caribbean sponge *Pseudoceratina crassa. Tetrahedron*, **1991**. *47*(10/11): p. 1809-1814.
21. Manzo, E., et al., Ceratamines A and B, antimitotic heterocyclic alkaloids isolated from the marine sponge *Pseudoceratina* sp collected in Papua New Guinea. *Org. Lett.*, **2003**. *5*(24): p. 4591-4594.
22. Kobayashi, J., K. Honma, and M. Tsuda, Lipopurealins D and E and purealidin H new bromotyrosine alkaloids from the okinawan marine sponge *Psammplysilla purea. J. Nat. Prod.*, **1995**. *58*(3): p. 467-470.
23. Tsuda, M., et al., Purealidins E-G new bromotyrosine alkaloids from the Okinawan marine sponge *Psammaplysilla purea. J. Nat. Prod.*, **1992**. *55*(9): p. 1325-1327.
24. Longeon, A., M. Guyot, and J. Vacelet, Araplysillins-I and-II: Biologically active dibromotyrosine derivatives from the sponge *Psammaplysilla arabica. Experientia*, **1990**. *46*(5): p. 548-550.
25. Mohanty, I., et al., Presence of bromotyrosine alkaloids in marine sponges is independent of metabolic and microbiome architectures. *msystems*, **2021**. *6*(2): p. 1-17.
26. Kernan, M.R. and R.C. Cambie, Chemistry of sponges, VII. 11, 19-dideoxyfistularin 3 and 11-hydroxyaerothionin, bromotyrosine derivatives from *Pseudoceratina durissima. J. Nat. Prod.*, **1990**. *53*(3): p. 615-622.
27. Tsukamoto, S., et al., Ceratinamine: An unprecedented antifouling cyanoformamide from the marine sponge *Pseudoceratina purpurea. J. Org. Chem.*, **1996**. *61*: p. 2936-2937.
28. Acosta, A.I. and A.D. Rodriguez, 11-Oxoaerothionin A Cytotoxic Antitumor Bromotyrosine-Derived Alkaloid from the Caribbean Marine Sponge *Aplysina lacunosa. J. Nat. Prod.*, **1992**. *55*(7): p. 1007-1012.
29. Abou-Shoer, M.I., et al., Bioactive brominated metabolites from the Red Sea sponge *Suberea mollis. J. Nat. Prod.*, **2008**. *71*: p. 1464-1467.
30. Rogers, E.W. and T.F. Molinski, Highly polar spiroisoxazolines from the sponge *Aplysina fulva. J. Nat. Prod.*, **2007**. *70*: p. 1191-1194.
31. Xynas, R. and R.J. Capon, Two New Bromotyrosine-Derived Metabolites From an Australian Marine Sponge, *Aplysina* sp. *Aust. J. Chem.*, **1989**. *42*: p. 1427-1433.
32. Rama Roa, M., U. Venkatesham, and Y. Venkateswarlu, Two bromo compounds from the sponge *Psammaplysilla purpurea. Ind. J. Chem.*, **1999**. *38B*: p. 1301-1303.
33. Venkateshwar Goud, T., et al., Two new bromotyrosine derived metabolites from the sponge *Psammaplysilla purpurea. Chem. Pharm. Bull.*, **2003**. *51*(8): p. 990-993.
34. Rogers, E.W., et al., Stereochemical Heterogeneity in Verongid Sponge Metabolites. Absolute Stereochemistry of (+)-Fistularin-3 and (+)-11-epi-Fistularin-3 by Microscale LCMS-Marfey's Analysis. *J. Nat. Prod.*, **2005**. *68*: p. 891-896.
35. Kijjoa, A., et al., Dibromotyrosine derivatives, a maleimide, aplysamine-2 and other constituents of the marine sponge *Pseudoceratina purpurea. Z. Naturforsch.*, **2005**. *60b*: p. 904-908.
36. Compagnone, R.S., et al., 11-Deoxyfistularin-3, a New Cytotoxic Metabolite from the Caribbean Sponge *Aplysina fistularis insularis. J. Nat. Prod.*, **1999**. *62*: p. 1443-1444.

37. Ragini, K., et al., Enantiodivergence in the Biosynthesis of Bromotyrosine Alkaloids from Sponges? *J. Nat. Prod.*, **2017**. *80*(1): p. 215-219.
38. Hirano, K., et al., Ma'edamines A and B, Cytotoxic bromotyrosine alkaloids with a Uinque 2(1H)Pyrazinone ring from sponge *Suberea* sp. *Tetrahedron*, **2000**. *56*: p. 8107-8110.
39. Shaala, L.A., et al., Subereamolline A as a potent breast cancer migration, invasion and proliferation inhibitor and bioactive dibrominated alkaloids from the Red Sea sponge *Pseudoceratina arabica. Mar. Drugs*, **2012**. *10*(11): p. 2492-2508.
40. Taki, A.C., et al., High-throughput phenotypic assay to screen for anthelmintic activity on *Haemonchus contortus. Pharmaceuticals*, **2021**. *14*(7): p. 616.
41. Preston, S., et al., Low cost whole-organism screening of compounds for anthelmintic activity. *Int. J. Parasitol.*, **2015**. *45*(5): p. 333-343.
